# Monitoring Electrochemical Dynamics through Single-Molecule Imaging of hBN Surface Emitters in Organic Solvents

**DOI:** 10.1021/acsnano.4c07189

**Published:** 2024-09-25

**Authors:** Eveline Mayner, Nathan Ronceray, Martina Lihter, Tzu-Heng Chen, Kenji Watanabe, Takashi Taniguchi, Aleksandra Radenovic

**Affiliations:** † Laboratory of Nanoscale Biology, Institute of Bioengineering Ecole Polytechnique Federale de Lausanne, EPFL STI IBI-STI LBEN BM, Lausanne CH-1015, Switzerland; ‡ Institute of Physics, Bijenicka 46, Zagreb HR-10000, Croatia; § Research Center for Electronic and Optical Materials, National Institute for Materials Science, 1-1 Namiki, Tsukuba 305-0044, Japan; ∥ Research Center for Materials Nanoarchitectonics, National Institute for Materials Science, 1-1 Namiki, Tsukuba 305-0044, Japan

**Keywords:** electrochemistry, single-molecule localization microscopy, 2D materials, spectral characterization, hBN

## Abstract

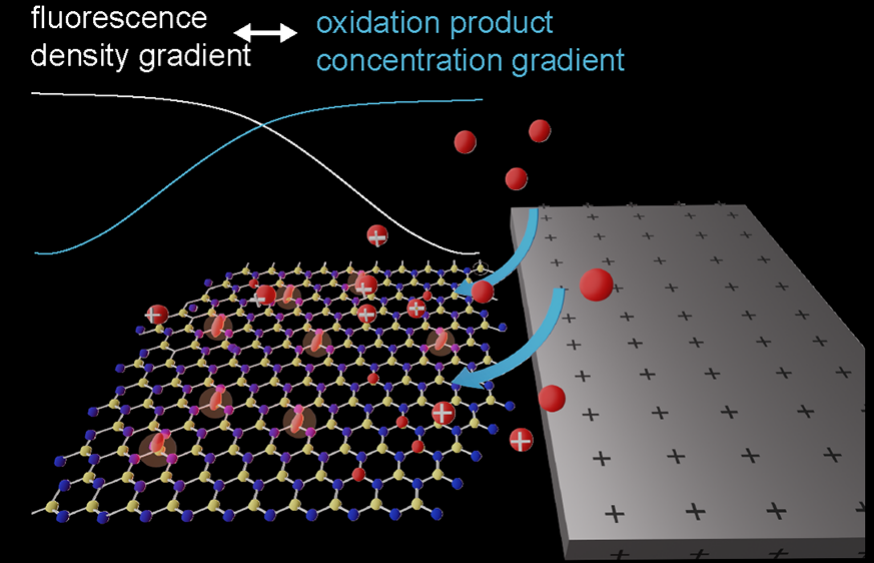

Electrochemical techniques conventionally lack spatial resolution and average local information over an entire electrode. While advancements in spatial resolution have been made through scanning probe methods, monitoring dynamics over large areas is still challenging, and it would be beneficial to be able to decouple the probe from the electrode itself. In this work, we leverage single molecule microscopy to spatiotemporally monitor analyte surface concentrations over a wide area using unmodified hexagonal boron nitride (hBN) in organic solvents. Through a sensing scheme based on redox-active species interactions with fluorescent emitters at the surface of hBN, we observe a region of a linear decrease in the number of emitters against increasingly positive potentials applied to a nearby electrode. We find consistent trends in electrode reaction kinetics vs overpotentials between potentiostat-reported currents and optically read emitter dynamics, showing Tafel slopes greater than 290 mV·decade^–1^. Finally, we draw on the capabilities of spectral single-molecule localization microscopy (SMLM) to monitor the fluorescent species’ identity, enabling multiplexed readout. Overall, we show dynamic measurements of analyte concentration gradients on a micrometer-length scale with nanometer-scale depth and precision. Considering the many scalable options for engineering fluorescent emitters with two-dimensional (2D) materials, our method holds promise for optically detecting a range of interacting species with exceptional localization precision.

Nanomaterials have emerged as promising high-performance sensors, offering exceptional sensitivity, temporal response, selectivity, and robustness.^1^ One of the best-studied nanomaterial-based sensors is the nitrogen-vacancy (NV) center in nanodiamonds, which has fluorescence sensitive to temperature,^2^ pH,^3^ strain,^4^ and electric/magnetic field.^5−8^ However, the precise engineering of NV centers in nanostructures remains challenging, and emission is sensitive to the crystallographic orientation and the distance to the surface. Alternatively, the atomic flatness and “surface-only” nature of 2D materials alleviate these difficulties while retaining robustness. In this study, we propose using hexagonal boron nitride (hBN), a wide band gap two-dimensional (2D) material with high thermal, mechanical, chemical, and optical stability to measure interfacial reactions in organic solvents.^9,10^

Single molecule or spatially resolved electrochemistry typically relies on scanning with a probe (electrochemical atomic force microscopy, scanning electrochemical microscopy, scanning tunneling microscopy, and scanning electrochemical cell microscopy).^11,12^ While scanning probe methods can provide detailed information about single molecules, including the formation of bonds,^13−15^ they are limited in field of view and prohibit sensing in confinement. High spatiotemporal resolution can be enabled by micro- or nano-arrays, but fabrication methods are often intensive, multistep, and expensive.^16,17^ Optical methods are an attractive solution to this problem, but nonfluorogenic redox pairs are difficult to study. Recently, electrochemical control over Alexa 647 fluorophore blinking was demonstrated^18^ and was shown to be beneficial for super-resolution/STORM microscopy.^19^ Conversely, in this work, we show that fluorescent emitters enable a local readout of electrochemical reactions occurring in their vicinity. Using label-free optoelectrochemical measurements of an unmodified 2D material, our method retains spatial sensitivity while harnessing a large field of view and fast temporal resolution. These factors are particularly relevant to heterogeneous samples, determining analyte concentration gradients and dynamically tracking analyte concentrations.

hBN’s band gap of ∼6 eV, makes it transparent to visible light. Still, defects in the atomic lattice create highly reactive centers and localized intrabandgap states that absorb and emit light in the visible range.^20−23^ While defects in 2D materials can be considered detrimental, in hBN they warrant applications of their own due to their environmental sensitivity: electrical control of hBN quantum emitters has already demonstrated in van der Waals heterostructures^24−26^ and the ODMR and photoluminescence (PL) of hBN defects were observed to change in response to temperature and magnetic field.^27^ Though most experimental work is completed in air or in vacuum, recent works have demonstrated oxygen-plasma-related defect fluorescent sensitivity to acidity in water,^28^ boron vacancy defects’ spin relaxation time dependence on the paramagnetic environment,^29^ and DNA interaction with unmodified and modified hBN.^30,31^

Recently, we reported that organic solvents activate single-photon emitters at the surface of unmodified BN.^32^ Our current work presented here showcases electrochemical modulations of these emitters, facilitating a spatially resolved readout of the activity of neighboring electrodes. Using spectral single molecule localization microscopy (SMLM), we monitor the electrochemically induced optical changes at the solid–liquid interface, which directly report on analyte dynamics, including diffusion and reaction kinetics. The methodology relies on the read-out of probes whose position is localized with nanometric resolution and over several micrometer ranges. By varying the potential difference of neighboring electrodes, we modulate the concentrations of analytes interacting with hBN, demonstrating the prospect of sensing trace species, possibly more reaction-specific than standard current tracing with a potentiostat. Several electrode configurations were tested using two- and three-electrode systems. Finding that the electric field could not be the source of modulation, we focused on the alteration of concentrations of species in the solvent and proposed that the emitter’s density modulation is based on the changing concentration of a quencher in solution.

## Results and Discussion

**Figure 1:**
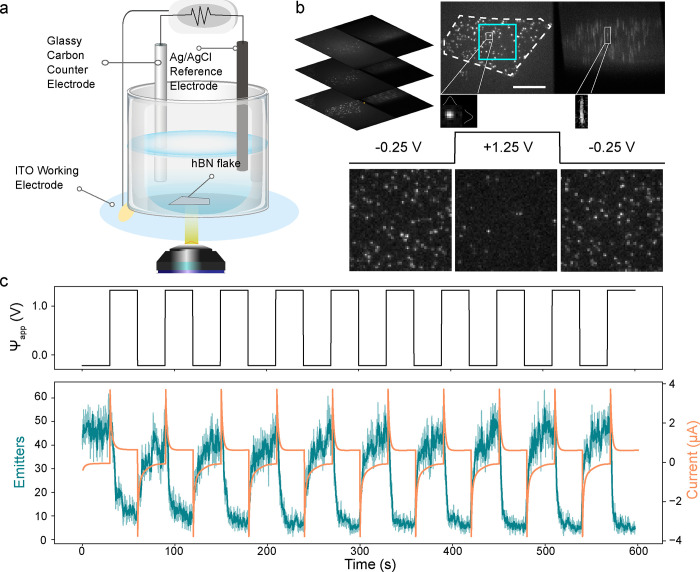
Acquisition scheme for opto-electrochemical measurements using hBN in organic solvents. (a) Simplified schematic of the opto-electrochemical setup in the out-of-plane three-electrode configuration. Inverted widefield microscope with a high NA objective is used to observe ensembles of hBN solvent emitters with the electrochemical chamber mounted above. (b) Frames of an hBN flake are acquired while changing the electrochemical potential of the surrounding ITO electrode. Only uniform areas (marked in teal) are used for the detection and localization of emitters, meaning edges and creases of hBN flakes are excluded. The scale bar is 5 μm. Emitted light is split into two channels: spatial (2D Gaussian emitters) and spectral (spread emission after passing through prism). Spatial channel shows the modulation of active emitters by the electrochemical bias in methanol. Several prototypical frames acquired with 30 ms frame rate are shown while switching between −0.25 V and +1.25 V vs Ag/AgCl, demonstrating the clear decrease in the number of active emitters when a positive voltage is applied. (c) In the spatial channel, emitter counts are time-correlated with the applied waveform and are used to show reversibility and quantify electrochemical effect. Response to 30 s pulses can be seen. Raw data is shown with 0.5 transparency and data smoothed with rolling average of 20 frames is shown in dark teal. The corresponding current trace is shown in orange.

Fluorescence was monitored in situ using a spectral SMLM^33^ configured with a home-built electrochemical cell (Figure 1a, details in Supporting Information, Figure S1), enabling high-resolution widefield imaging (see Materials and Methods). Three different electrode configurations were used to narrow the mechanism involved. These configurations will be referred to based on the orientation of the electric field applied to the hBN: out-of-plane, in-plane, and stray. While hBN is nonconductive and thus cannot act as an electrode itself, flakes were exfoliated from the bulk and placed in proximity to working electrodes (indium tin oxide, i.e., ITO or titanium), and the electrochemical potential of these electrodes was modulated. The general acquisition scheme (Figure 1) is as follows: image stacks are acquired (typically 15–50 ms per frame), while electrochemical potential is controlled. Spatial and spectral channels are read-out onto the EMCCD chip, and a uniform region of the flake is isolated. Emitters are localized (see Materials and Methods) to provide information on counts, spectra, and residence times. The trajectories of emitters were also determined, but their distribution remained isotropic in all field configurations. The example frames show hBN emitters in methanol optically responding to a +1.25 V pulse at the surrounding ITO electrode vs Ag/AgCl. The change in number of emitters can easily be observed (Figure 1b,c) at high changes in applied electrochemical potential. The applied electrochemical potential is denoted as Ψ_app_*=*Ψ_w_*–*Ψ_ref_: the potential at the working electrode versus the potential at the reference electrode.

**Figure 2:**
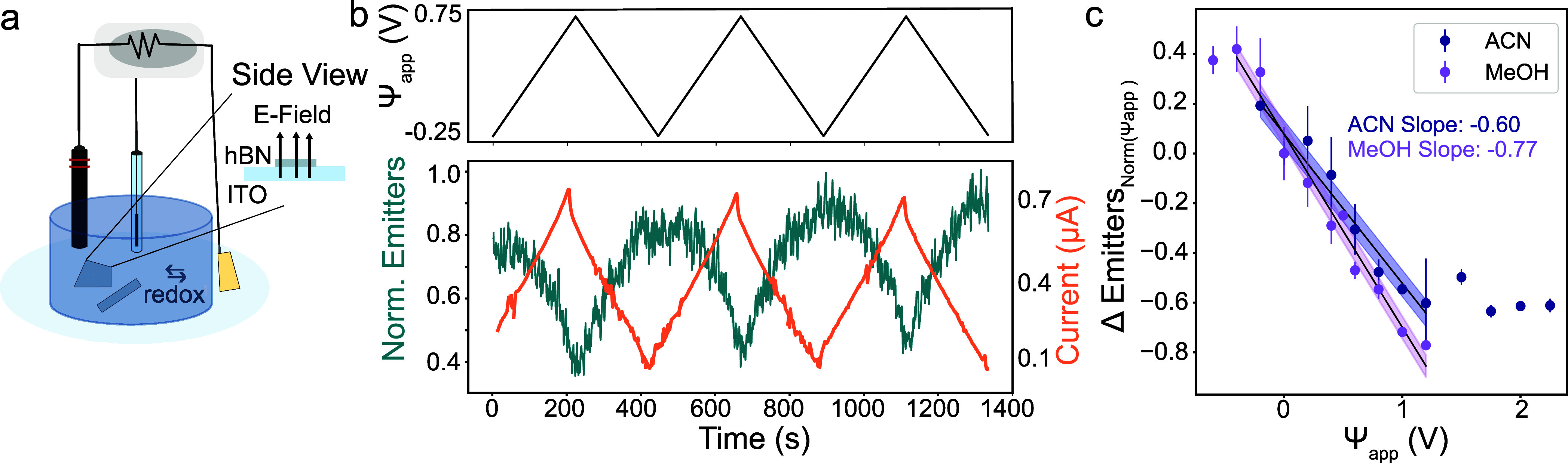
Out-of-plane opto-electrochemical measurement with varying applied potential. (a) Simplified schematic of the three-electrode out-of-plane measurement configuration with the inset side view showing the field orientation relative to the flake. hBN flakes are transferred to the ITO-coated coverslip, which is in contact with the solvent above. Glassy carbon electrode serves as a counter electrode and a thin leakless Ag/AgCl electrode as the reference electrode. (b) Top panel shows the potential vs Ag/AgCl (Ψ_app_) applied to the ITO working electrode with a triangular waveform for over 20 min in acetonitrile. Bottom panel shows the resulting normalized emitter counts (teal) and current (orange). (c) Change in number of emitters was quantified for various Ψ_app_ using different flakes in methanol and acetonitrile (full data in Supporting Information, Figure S2). Change was always characterized relative to that specific experiment’s mean binned emitter counts at Ψ_app_ = 0 V. Linear decrease in the number of emitters per frame is seen at increasingly positive voltages from a Ψ_app_ of −0.4 V and then the delta plateaus. Standard errors for each potential are shown as bars for individual points and as shading for the linear fit.

In the out-of-plane electrochemical configuration, thick (several tens to a few hundred layers) hBN flakes were exfoliated onto glass coverslips coated with a layer of ITO, as shown in Figure 2a. The thickness of the flake was not controlled but can be roughly gauged by optical contrast. Flake thickness showed no impact on measurement, as is intuitive with a surface-mediated mechanism. The ITO (∼70 nm thick) served as the working electrode in a three-electrode configuration while remaining transparent enough to retain a high photon capture. The glassy carbon counter electrode and leakless Ag/AgCl reference electrode were suspended in the solvent above the focal plane. Emitters were excited using a 561 nm laser (power density ∼1.6 kW·cm^–2^ unless otherwise specified), and the fluorescent signal was collected through the same objective. A potentiostat was used to apply the potential to the ITO working electrode vs Ag/AgCl reference.

The number of active emitters per frame was monitored using the spatial channel while varying the potential applied to the ITO working electrode versus the Ag/AgCl reference. Voltage waveforms varied in shape (triangular and square), magnitude, and time duration. An example of a triangular waveform applied in acetonitrile is shown in the top panel of Figure 2b and the corresponding current in the bottom panel. The impact of this potential can be seen on the normalized number of emitters per frame, which follows inversely the shape and magnitude of the current. The traces also show that emitter quenching during cycling is reversible, providing evidence that the involved redox reactions are reversible, although the forward and backward reactions may occur at different rates.

Square voltage pulses were also applied in methanol and acetonitrile to quantify the change in emitters against the changing potential. Figure 2c derives from the response of different flakes to a variety of square pulse patterns. While the normalized number of emitters reported generally normalizes the maximum number of emitters in that cycle to one, average changes in emitter counts were calculated relative to the number of emitters at Ψ_app_ = 0 V. We define the change as . The mean and standard deviation of each change were determined from the binned counts at each voltage step (the data for both solvents are shown in Supporting Information, Figure S2). Ψ_app_ = 0 V has a nonzero standard deviation because not every 0 V voltage step of each cycle has the same binned number of emitters, and the value fluctuates around the mean. While different rates of change between solvents can be observed, the behavior is consistent: at increasingly positive potentials and higher anodic currents, the number of active emitters decreases, while at increasing negative potentials, the number of active emitters increases. This inverse relationship can be attributed to a change in concentration of species involved in activating or quenching the hBN emitters. At potentials greater than or equal to 1.5 V, the change in number of emitters plateaus in acetonitrile. Although this can only be observed in acetonitrile, it is likely this would also hold true in methanol, but the working range is more limited in this solvent (Supporting Information, Figure S3). The slight decrease at Ψ_app_ = −0.6 V in methanol (Figure 2c) can be understood due to the instability of ITO at this potential in organic solvents, and higher magnitude negative potentials were not probed in this configuration for this reason. This limited stability has been previously documented,^34,35^ and the stable range for various organic solvents used in this paper was determined by cyclic voltammetry (CV available in Supporting Information, Figure S3). Note that using this stable region does not preclude all reactions from occurring on a smaller scale but rather avoids large-scale reactions of the solvent or electrode. We suspect that the linear rise in current against potential seen in the smaller scale CV in Figure 2b is due to a mix of capacitive and Faradaic currents, with the Faradaic current owing to quencher reactions and occurring on a small magnitude due to the low concentration of reactions, as compared to the greater current scale of the methanol CV, as shown in Supporting Information, Figure S3.

In the out-of-plane orientation, the origin of the optical modulation was elucidated by passivating the surface with a thin insulating layer of aluminum oxide (100 nm). While the electric field remained consistent in orientation and magnitude, this passivation effectively suppressed the analyte redox reaction, leading to the suppression of the response of emitters. Therefore, we could conclude that the emitter response originates from chemical species diffusing from the electrodes, resulting in changes in concentration during cycling. The thin film characterization via ellipsometry and voltammetry results are available in Supporting Information, Figure S4.

The dependence of the signal on the distance to the electrode was first examined using a “stray-field” configuration, wherein the ITO electrode was replaced by a thin titanium electrode patterned onto a glass slide (discussion on stray-fields, Supporting Information, Figure S5). The signal modulation was consistent with the out-of-plane measurements, despite the change in orientation of the field and change in electrode, while the magnitude of the effect was influenced by distance to the electrodes. This micrometer distance dependence is a useful characteristic for a high spatial resolution sensor. Although we probed inside the working potential window of ITO, the change of electrode material also demonstrates that the decrease in emitter activity near positive electrodes was not due to ITO electrode deterioration– ITO is an *n*-type semiconductor, so at high positive potentials, its conductivity and stability decreases.^36^

### Examining Reaction Kinetics and Spectra

We then turned to the applicability of our optical method to report on dynamic changes, specifically, focusing on anodic reaction kinetics. The reaction at the anode is hypothesized to be the oxidation of trace water and will be discussed further in the proposed mechanism and the role of water and H^+^ section. Since the Faradaic current in an electrochemical system is by definition proportional to the reaction rate at the electrode surface by eq 1
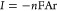
1where *I* is the current, *n* is the number of electrons transferred, *A* is the area, *F* is the Faraday constant, and *r* is the reaction rate. This means that for an anodic reaction, the current density is described by eq 2(37)
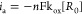
2where *i*_a_ is the anodic current density in A·m^–2^, *k*_ox_ is the oxidation rate constant, and [*R*_0_] is the surface concentration of the reductant. Since electrochemical currents are potential-dependent, it follows that the rate constant is also potential-dependent. Although the reaction would occur at the ITO electrode, optical modulation of the hBN emitters provides another means of determining the reaction rate constants, given that our analyte reaction occurs proportionally to the bulk current. In our case, we can analyze ensemble emitter dynamics while applying pulses of positive potentials to the ITO electrode.

While Tafel analysis is most commonly applied in the context of electrocatalysts, it is applicable to any electrochemical reaction to inform on rate-limiting steps.^37^ However, one should note that Tafel analysis does have significant limitations. For example, it cannot differentiate two mechanisms that share the same expected Tafel slope.^38,39^ In our case, the slope can be influenced by any step of the reaction on ITO: the reaction between emitter and quencher, the diffusion of the reactant to the electrode, or the reaction at the electrode surface, which can be complex and rate-limiting.

The Tafel slope is derived at high anodic overpotentials from the simplified Butler–Volmer equation, which leads to a simple relationship between the overpotential, η, and the current by eq 3
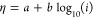
3where *b* is the Tafel slope. The overpotential is defined as the difference between the electrode potential, *E*, and the standard potential, *E*_0_′ (the half-cell potential). Applied potential was used instead of overpotential, but since *E*_0_′ is a constant, it does not impact the derived slope.

**Figure 3:**
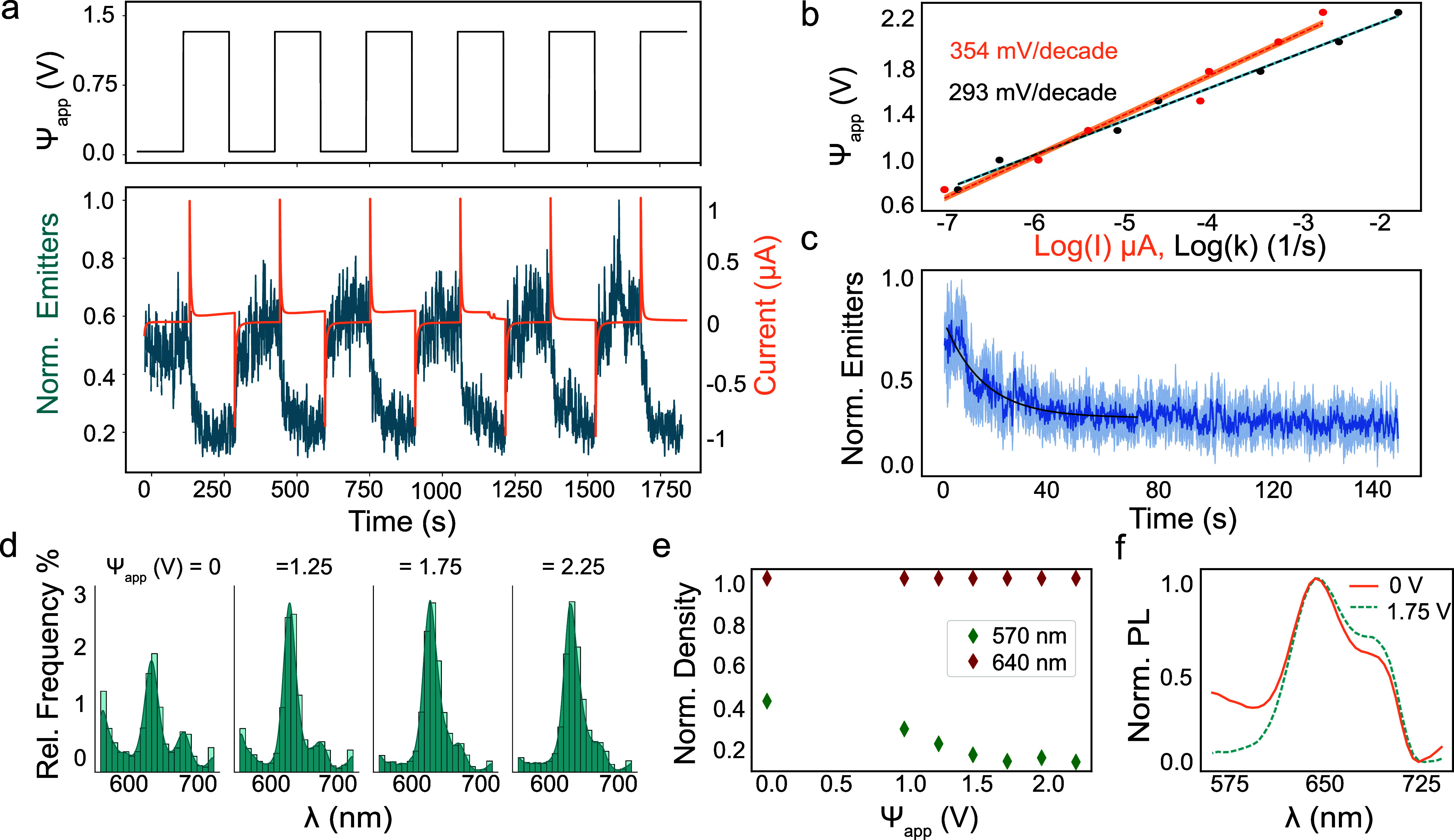
Out-of-plane localization response characteristics. (a) Long square pulses (150s) were applied cyclically in acetonitrile while the localizations were recorded. Current response to Ψ_app_ = 1.5 V pulses is shown in orange, while the response of the normalized number of emitters, is shown below in teal. (b) Tafel plot obtained from steady state currents recorded during the pulsing experiments shown in part a. Tafel slope derived from a linear fit of these seven points is indicated by the black dashed line and standard deviation of fit in light blue. Orange dashed line indicates linear fit with shadowed standard deviation for the emitter-derived slope. (c) Example decay of emitters in response to a positive pulse averaged from the 5 pulses shown in a. Dark blue line shows the mean number with a light blue cloud of the standard deviation. (d) The kernel density estimation (KDE) overlaid on the histogram of spectral peaks of single emitters at various voltages in acetonitrile, obtained using our sSMLM. Characteristic main peak around 640 nm consistently leads. (e) From the histogram, two main peaks (570 and 640 nm) relative prominences are plotted. Main group at 640 is constant stray spectral groups are reduced at increasing voltages. (f) Average sSMLM-obtained spectra of all emitters at Ψapp = 1.75 V in acetonitrile. From fitting the averaged PL spectra at Ψ_app_ = 1.75 V, we find the zero-phonon line to be 643 nm and the phonon sideband to be 687 nm.

Long pulses were applied to the working electrode in the three-electrode out-of-plane configuration at increasingly positive potentials for seven different potentials using an acquisition time of 15 ms over a 9 × 9 μm^2^ area (example trace in Figure 3a). The reaction kinetics derived from the potentiostat-reported current were compared to those derived using the emitter temporal dynamics (Figure 3b). Smaller Tafel slopes mean that the reaction follows faster kinetic processes because the same currents can be achieved at lower overpotential. Although Tafel slopes generally relate to reaction kinetics rather than mass transport, differentiating between a slow reaction and a diffusion-limited reaction can be difficult. For example, the theoretical Tafel slope of the well-known hydrogen evolution reaction is 30 mV·dec^–1^ but experimentally reported slopes can reach over 290 mV·dec^–1^^37,40^ because the reaction is complicated, faces significant barriers, and/or has rates limited by diffusion.

The optically derived slope was determined by fitting the exponential decay behavior in emitter counts to first-order reactions and determining the kinetic rate constants. In Figure 3c, this decay is shown averaged from each pulse in Figure 3a. The reaction order was determined to be pseudo-first-order since the optically monitored reaction between emitter and quencher depends primarily on the quencher concentration, as indicated by the direct dependency of the rate on potential and by comparing the *R*^2^ goodness of fit for first- and second-order modeling (see Supporting Information discussion on Kinetics, Figure S6). The consistency in magnitude between the optically and electrochemically derived slopes validates the optical method for monitoring an electrochemical process part of the current and further that the analyte reaction faces significant barriers.

The spectra of single emitters were used to monitor the identity of the emitters at the same seven potentials in acetonitrile. The acquisition time was increased to 50 ms to increase the signal-to-noise ratio and to record the spectral channel. Spectra were obtained using the spectral channel of our sSMLM setup (Figure 1). The histogram and kernel density estimation (KDE) of spectra from single emitters are seen in Figure 3d at various voltages (20 bins, 0.75 binwidth smoothing). Plotting the density of different spectral groups from the histograms at increasing potentials (Figure 3e) shows that the group with zero-phonon line (ZPL) around 640 nm is consistently dominant, but that there is a pronounced effect of voltage on the spectral group with a peak around 570 nm, a group that has previously been observed on plasma-treated and CVD-grown hBN in water.^33^ The same purging effect on stray emitters is seen in the in-plane at high potentials (Supporting Information, Figure S7). With the observation of several types of emitters with distinct responses to voltage in the same field of view, we demonstrate the opportunity for multiplexing in optical electrochemistry. A video of switching between +1.5 and 0 V in acetonitrile that was used for spectral analysis (acquisition: 50 ms) is available as Supporting Information, Video S1.

Examining the spectral channel also revealed that the Stark effect does not play a significant role on the main emitter population. Figure 3f shows the result of averaging individual spectra at Ψ_app_ = 0 and 1.75 V. At 1.75 V, averaging essentially erases the presence of other edge groups, and plotting the ZPL and phonon sideband (PSB) shows no significant change (Supporting Information, Figure S8). Finally, by using subdiffraction localization and single molecule tracking techniques, the residence times of individual emitters were determined. Groups of long-lasting and short-lasting emitters emerged from the bulk at positive potentials, showing groups with distinct responses to potential (Supporting Information, Figure S9), demonstrating in residence time another opportunity for monitoring of distinct emitters in the same field of view.

### In-Plane Measurements Demonstrate a Spatially Responsive Electrochemical Sensor

**Figure 4:**
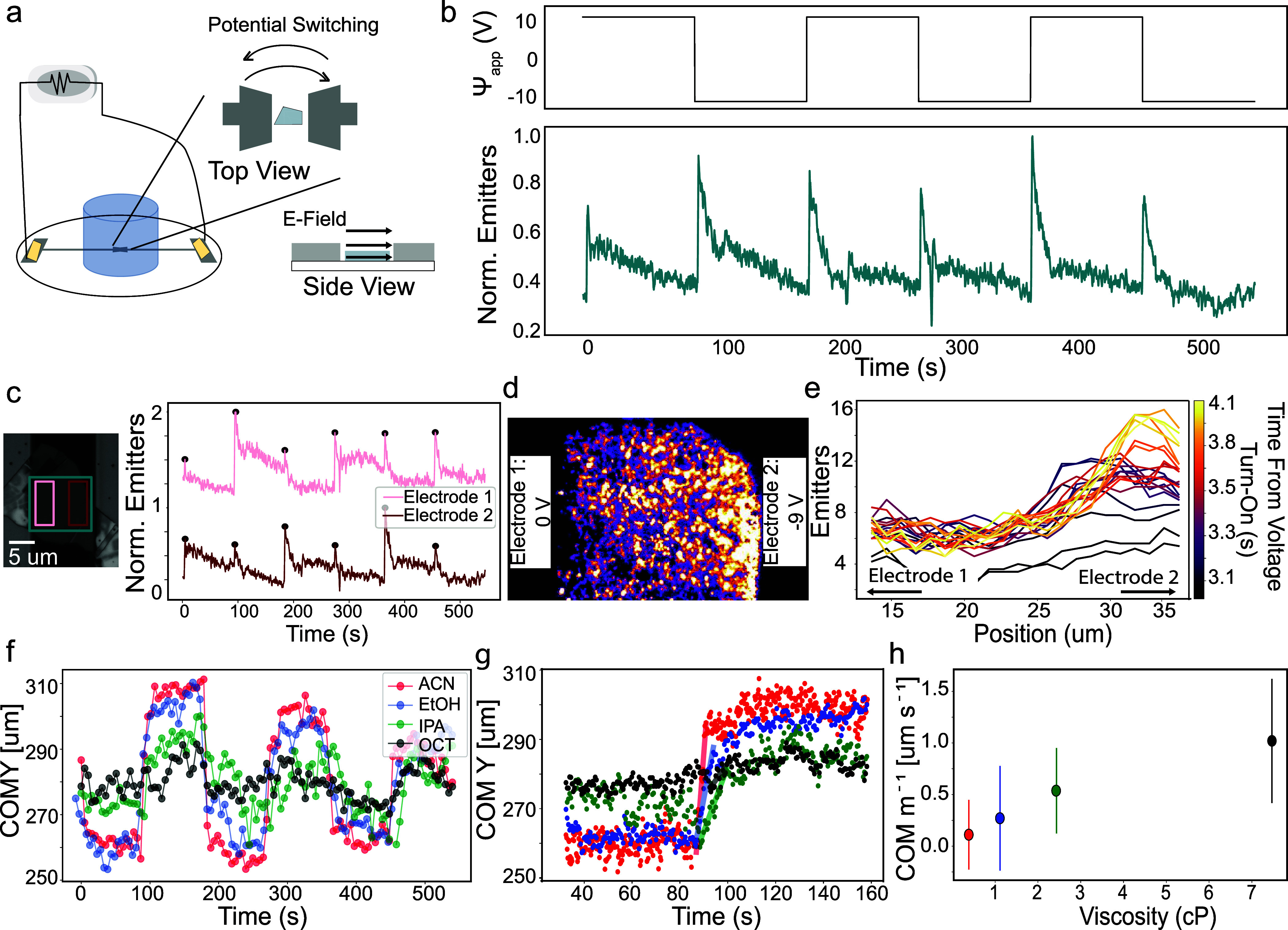
In-plane experiments show spatial modulation. (a) Simplified schematic of in-plane two-electrode configuration with the inset side view showing the field orientation relative to the flake. Patterned titanium electrodes are connected to the potentiostat via copper tape contacts that are isolated from the solvent. Top electrode served as the working electrode and bottom as counter/reference. (b) Potential was varied at the working electrode and the normalized number of localizations for the entire flake region is reported in teal over time (50 ms frame rate) for this example trace in acetonitrile. (c) Optical microscope image shows an example sample wherein a flake was transferred between two titanium electrodes. Localizations are analyzed by region, wherein the pink area (“electrode 1”) is near the working electrode and the red area (“electrode 2”) is near the counter/reference electrode. Normalized localizations are then reported with an offset of 1. (d) 50 frames were stacked together from one electrode polarization to show how the density of emitters visibly changes over the distance of the flake in response to the concentration of the analyte. Video of the switching occurring is available as Supporting Information, Video S2. (e) In acetonitrile, the concentration gradient after the voltage is applied in the first cycle can be seen building over time. Time is reported from the time of the application of the voltage and is shown with inferno color mapping. There is a delay from the onset and then the concentration changes over the course of ∼1 s. The position is reported as distance from the top of the frame in micrometers and the time is from the onset of the experiment (the first cycle). (f) The center of mass (COM) of the emitter localizations can be passed between the two electrodes by switching the polarization (as in part b) for acetonitrile (ACN), ethanol (EtOH), isopropyl alcohol (IPA), and octanol (OCT). Here, a position point is shown every 5 s (g) the region corresponding to a switch in polarizations, is averaged for the three pulses and the slope is determined for this linear switching region, corresponding to speed of switching. (h) Reciprocal of the COM slope (COM m^–1^) was plotted against the viscosity in centipoise (cP) for these four solvents.

Having characterized the emitter response using an isotropically surrounding electrode, we turned to an in-plane electrode configuration, wherein the distance from the active electrode surface could be controlled. We patterned two titanium electrodes onto glass coverslips (depicted in Figure 4a) using a custom silicon/silicon nitride shadow mask and electron-beam evaporation (see Materials and Methods for details). hBN flakes were then deterministically placed between these electrodes. The electrodes were oriented such that there was a blank strip 20 to 40 μm wide, bisecting the titanium layer and creating two large pads. The top titanium electrode was connected to the working electrode, and the bottom electrode to the counter/reference electrode. With the flake in symmetric contact with both electrodes, electrode polarization was switched over time, effectively switching the positions of the positive and negative electrode with each pulse. The total emitter counts responded the same way in response to pulses, regardless of the polarization direction. In acetonitrile, emitter counts spiked initially at each pulse and fell to equilibrium during the 90 s pulses (example in Figure 4b); this can be understood as a capacitive spike occurring in the movement of ions in solution. Other contact orientations were also tested and are reported in Supporting Information, Figure S10, and show contact plays no role except for due to the inherent distance from electrode changes.

Emitter density was found to follow the charge of the electrodes as they were switched. The emitter density increased near the more negatively charged electrode (Figure 4c,d) while it decreased near the more positively charged electrode, which indicated that we were able to simultaneously monitor both redox half reactions in the same field of view. The video corresponding to this data is available as Supporting Information, Video S2, and shows the emitters responding to the switching of electrodes 1 and 2 and an image from the opposite polarization as Figure 4d and is available in Supporting Information, Figure S11. Concentration gradients can even be seen changing in time, with the change in analyte profile in acetonitrile changing rapidly over ∼1 s (Figure 4e). To establish the generality and examine the influence of solvent properties, we then repeated measurements using ethanol, isopropyl alcohol, and octanol (concentration profiles over time for other solvents are available in Supporting Information, Figure S11). The center of mass (COM), defined as the average *x* and *y* positions of all localizations in that frame, was tracked while the electrode polarization was switched for the three cycles (Figure 4f). The switching region of each solvent was fit linearly (Figure 4g). Finally, the reciprocal of the slope was plotted as a function of the viscosity of solvent (Figure 4h). As is understood by Fick’s Law, the rate of diffusion is directly proportional to diffusivity (the inverse of viscosity), so increasing solvent viscosity results in slower diffusion. Because the COM switching rate was found to be inversely related to the viscosity, it follows that either the rate of movement from the electrode to the flake of the redox analyte was limited by diffusion or that the rate-limiting step in the reaction at the electrode was limited by diffusion. Since we observed high Tafel slopes between current- and emitter-derived kinetics, a hypothesis of diffusion-limited reaction remains reasonable, albeit likely involving multiple factors.

### Proposed Mechanism and the Role of Water and H^+^

Through the testing of different electrode configurations, we were able to reject an emitter modulation mechanism based on electric field or charge transfer and to propose a redox-active analyte-based quenching mechanism. Because the modulation is consistent across different solvents and the onset of the effect on emitters is not correlated with the onset potential of a reaction of the bulk solvents (Supporting Information, Figure S2), we consider that there is a common trace species present in the solvents, which is modulating the emitter density by quenching. Based on our observation, we propose that the quencher may be H^+^, originating from the oxidation of reactive trace water existing in the solvent. We have also demonstrated the existence of two spectral groups. One group, centered around 570 nm, has been previously observed in oxygen plasma-treated hBN images in the presence of water^33^ and exhibits notably rapid quenching under increasing potential (Figure 3d).

The proposed proton-based mechanism was demonstrated by the deliberate introduction and removal of water and H^+^ (in the form of HCl) to otherwise pure organic solvents (Supporting Information, Figure S13). Our work shows that as the concentration of water increased, the number of active emitters dropped. Removing water traces with a molecular sieve also removed the effect of voltage cycling at potentials that had a strong impact on “wet” acetonitrile (Supporting Information, Figure S14), indicating that water plays an important role in the modulating reaction mechanism. We also showed that when introducing H+ through HCl, the density of emitters fell more than 25 times faster than through introducing water (Supporting Information, Figure S13). This not only supports the proposed mechanism but also shows that fluorescent emitters in our system are highly sensitive to the H^+^ content in organic solvents. Measuring H^+^ content would be highly relevant in methanol fuel cells,^41,42^ and using our spatially sensitive system, the concentration could be monitored as a function of distance from electrodes in time.

As is schematically illustrated in Supporting Information, Figure S15, we propose that the emitter density is modulated by the electrochemical reduction and oxidation of a quencher at the two electrodes in the field of view. Although water oxidation typically occurs at higher overpotentials and the concentration of water would be low (coming from initial presence in purchased solvents^43^ and exposure to the environment) mixing water with acetonitrile has been shown to create water clusters with increased susceptibility to oxidation due to the modification of its molecular aggregation behavior.^44^ Water is a known quencher of red emitting dyes,^45^ and it has been suggested that this is due to excited state proton transfer from water to emitter.^46,47^ Given this adventitious source of water and H^+^, the mechanism of quenching could be understood by proton quenching of the hBN-solvent emitter. However, in terms of modulating mechanisms, we acknowledge other possible routes involving reactions of the solvent itself and discuss them in Supporting Information discussion on mechanisms. While our findings leave room for more thorough investigations into the quenching mechanism and emitter identity, we nonetheless clearly demonstrate that the quenching is triggered by electrochemical reactions, providing an optical method for monitoring analyte species that can be applied to other systems with modified 2D materials.

## Conclusions

In this article, we describe the use of single molecule fluorescence microscopy with unmodified hBN to optically monitor electrochemical reactions and analyte concentrations in time. We also demonstrate the ability for local analyte concentration and diffusivity measurements in confinement and locations that a scanning probe cannot access. hBN emitters were shown to report on electrochemical reactions occurring in their vicinity as well as concentrations of water and H^+^. The electrochemical measurement configurations herein presented allowed us to narrow down the mechanism to an electrochemical modulation, ruling out other possible mechanisms such as modulation by charge injection or electric field/orientation. The spatial resolution inherent in the surface-only nature of the hBN and the localization microscopy scheme make it a favorable method for sensing reactions with high spatial specificity, with nanometer resolution in *z* and micrometers in *x* and *y*. The temporal resolution also enables us to report on changes in concentration over tens of milliseconds. With these attributes, it is possible to monitor diffusion and the buildup of a concentration gradient in time. Our experiments clearly demonstrate the benefits of integrated opto-electrochemistry. Specifically, we show spatially resolved electrochemical measurements with a large field of view, an approach that holds promise for probing electrochemistry in nonuniform materials and in confinement. Additionally, the work highlights the advantages of using 2D materials in sensing, which offer additional engineering opportunities via defect creation or surface modification, facilitating the monitoring of other specific reactions.

## Materials and Methods

### Out-of-Plane Sample Preparation

ITO-coated coverslips (70–100 Ohms Sq^–1^, 25 mm diameter *x* 0.17 mm, #1.5 thick coverslips) were purchased from Diamond Coatings Ltd. The coverslips were cleaned by sonication in acetone then IPA, and finally with oxygen plasma for 90 s with 100 W. High-quality hBN flakes were gently exfoliated from bulk crystals^48^ using low-adhesion Nitto tape and transferred onto the ITO coverslips. The thickness of the flake was not controlled but could be roughly gauged by optical contrast. This showed no impact on the measurement, as intuitive with a surface-mediated mechanism.

### Electrochemical Methods

An electrochemical cell compatible with SMLM was designed for a 25 mm coverslip with three possible configurations, as named for the orientation of the electric field relative to the hBN flake: out-of-plane, in-plane, and stray-field. The cell was then machined from PEEK, which was highly resistant to organic solvents. A 3D rendering of the cell is available in Supporting Information, Figure S1.

Cyclic and pulse voltammetry experiments were carried out using a PalmSens4 instrument configured in a three-electrode (out-of-plane and stray-field) or two-electrode (in-plane) configuration. PTFE encapsulated glassy carbon and ITO were used as the counter and working electrodes, respectively. An Ag/AgCl leakless electrode suitable for organic solvents (Alvatek) was used as the pseudo reference electrode. Measurements were carried out at room temperature, and any openings to the environment were covered but not sealed in an airtight manner from the environment. A new cell, which had every opening (for electrodes and viewing windows) sealed from the environment, was made for the anhydrous experiments shown in Supporting Information, Figure S14. Scan rates of CV varied from 7.5 to 50 mV·s^–1^. The potentials are all reported against the leakless Ag/AgCl electrode in the three-electrode configuration. In the two-electrode configuration, the counter and reference electrodes were the same titanium electrode.

### In-Plane + Stray-Field Sample Preparation.

Electrodes were patterned using a silicon/silicon nitride (Si/500 nm Si_3_N_4_) stencil masks and evaporation of titanium. The stencil mask was prepared by using positive photolithography with reactive ion etching and subsequent KOH etching. The distance between electrodes was retained by the silicon nitride membrane, which remained after isotropic KOH etching. Various electrode spacings were tested by varying the membrane width.

Glass coverslips were cleaned with acetone, IPA, and oxygen plasma before metal patterning with electron-beam evaporation. Various electrode heights (100–250 nm) and spacings (10–100 nm) were tested. All wafer fabrication and evaporation processes were performed in the clean room.

To place hBN flakes between (in-plane) or adjacent to (stray-field) patterned electrodes, a dry transfer method with a home-built transfer platform is used. A stamp, consisting of a thin film of polypropylene carbonate (PPC) spin coated on a mound of polydimethylsiloxane (PDMS) mechanically stabilized by a glass slide, is attached to a micromanipulator under a microscope. A Si_3_N_4_/Si substrate with hBN to be transferred is fixed onto a transfer stage, and the desired hBN flake is identified using optical microscopy and positioned with a micromanipulator. Any PPC residue is then washed using acetone and IPA. The mask lithography pattern and an optical image of an example cover slip with transferred hBN between titanium electrodes are available in Supporting Information, Figure S16.

### ITO Surface Passivation.

The ITO coverslips were passivated using atomic layer deposition (ALD) of 100 nm of alumina oxide (Al_2_O_3_). Characterization of the thin film was done using ellipsometry in 8 locations in the center 8 mm of the coverslip surface, the area in contact with the organic solvent during imaging (available in Supporting Information, Figure S3). A 3 mm diameter circle adjacent to the edge of the coverslip was isolated from coating during ALD via Kapton tape, which was then used for contacting the ITO to the potentiostat via the same silver-tape-cover wire to potentiostat configuration as previously described.

### Spectral Single Molecule Localization Microscopy

The samples were excited using a 561 nm laser (Monolithic Laser Combiner 400B, Agilent Technologies) collimated and focused on the back focal plane of a high-numerical aperture oil-immersion objective (Olympus TIRFM 100×, NA: 1.45). Excitation power was controlled on a range from 10 to 80 mW over a ∼ 2.5 × 10^3^ μm^2^ illumination spot resulting in a power density of 0.4–3.2 kW·cm^–2^. The sample was mounted in a PEEK electrochemical chamber placed on a piezoelectric scanner (Nano-Drive, MadCityLabs). Fluorescent emission is collected by the same objective, separated from excitation using the dichroic and band-pass emission filter (ZT488/561rpc-UF1 and ZET488/561m, Chroma), and finally projected on an EMCCD camera (Andor iXon Life 897) with an electron multiplying gain of 150. Exposure times ranged from 15 to 50 ms and stacks from 3 to 114 thousand frames.

Spectral SMLM is performed by splitting the emission via a beam splitter into two paths described in detail previously^33^ and summarized briefly here. Path 1, the spatial path, travels directly to the EMCCD, while path 2 passes through an equilateral calcium fluoride (CaF_2_) prism, which spreads the light (spectral path). Acquired image stacks from sSMLM were processed using ThunderSTORM.^49^ Emitter filtering and counting were performed using ThunderSTORM, omitting localizations that had intensity less than 300 photons or 30<σ_PSF_ < 250 nm.

For spectral processing, only emitters with intensity greater than 350 photons were used. The spectral channel was related to the spatial channel and wavelength via a facile matrix transform (*x*_SPEC_, *y*_SPEC_) = A × (*x*_LOC_, *y*_LOC_) + B, where A is a 2 × 2 matrix and B is a column vector. The position of different wavelengths (B vector) was calibrated using broadband beads with narrow bandpass filters. An example bead calibration is shown in Supporting Information, Figure S17. The single molecule spectra were determined by the peak intensity in the box of 6 pixels × 40 pixels centered at the transformed emitter position. In single molecule spectra, the highest intensity position corresponds to the peak wavelength of the emitter and the relative densities of each of these populations were determined using the KDE function of the Python library Seaborn with 20 bins and 0.75 binwidth smoothing (Figure 3d). Smoothed histograms with spectral SMLM data have previously been used to display population distributions of single molecule spectra.^50^ Average ensemble sSMLM spectra (shown in Figure 3f) were normalized and fit using two Lorentzians, corresponding to ZPL and PSB using the Python 3.7 package LMFIT42.^51^ The consistency in wavelength between the spectral population with the highest density and the average ensemble fit ZPL also shows the validity of the single molecule approach.

## Chemicals

All chemicals were purchased with high purity grade. No effect of the supplier on hBN fluorescence activation was observed.

## Supporting Information Available

In-plane and out-of-plane experiment videos, 3D rendering of electrochemical cell, working electrode characterization, discussion on reaction kinetics, in-plane electrode fabrication, in-plane spectra, additional experiments with water and HCl, and discussion on mechanisms (PDF)
(AVI)
(AVI)
(PDF)

## Supplementary Material

nn4c07189_si_001.pdf

nn4c07189_si_002.avi

nn4c07189_si_003.avi
