# Supplementary material for: Monitoring Electrochemical Dynamics through Single-Molecule Imaging of hBN Surface Emitters in Organic Solvents
Source: ACS Nano. 2024 Sep 25;18(40):27401–10. doi: 10.1021/acsnano.4c07189 (PMC11468151; doi:10.1021/acsnano.4c07189)
Supplement: Supplementary file 1 — nn4c07189_si_001.pdf [file nn4c07189_si_001.pdf]

Supplementary Information for "Monitoring Electrochemical Dynamics through Single-Molecule Imaging of hBN Surface Emitters in Organic Solvents"

Eveline Mayner[1], Nathan Ronceray[1], Martina Lihter[1,2], Tzu-Heng Chen[1], Kenji Watanabe[3], Takashi Taniguchi[4], Aleksandra Radenovic[1]*

[1] *Laboratory of Nanoscale Biology, Institute of Bioengineering Ecole Polytechnique Federale de Lausanne, EPFL STI IBI-STI LBEN BM, CH-1015 Lausanne, Switzerland*

aleksandra.radenovic@epfl.ch

[2]*Institute of Physics, Bijenicka 46, HR-10000 Zagreb, Croatia*

[3]*Research Center for Electronic and Optical Materials, National Institute for Materials Science, 1-1 Namiki, 305-0044 Tsukuba, Japan*

[4]*Research Center for Materials Nanoarchitectonics, National Institute for Materials Science, 1-1 Namiki, 305-0044 Tsukuba, Japan*

Contents

Supplementary Figures and Discussions 2

Supplementary Discussion on Stray-Fields 6

Supplementary Discussion on Kinetics 7

Supplementary Discussion on Mechanisms 13

Identifying and Excluding Candidate Reactant Species 13

Experiments Showing the Involvement of Water and H^+ 14

Proposed Electrochemical Mechanism 17

Captions For Supplementary Videos 19

Supplementary References 20

Supplementary Figures and Discussions

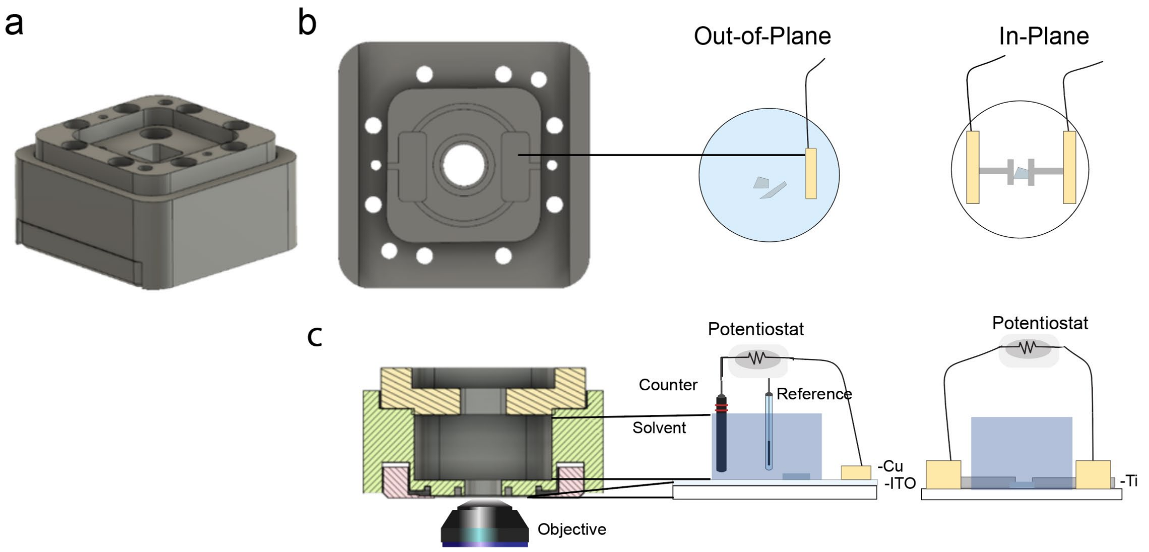

Supplementary Figure 1: 3D rendering of the electrochemical cell. a) The electrochemical cell compatible with our high-resolution imaging microscope is comprised of three parts that are screwed together for a tight seal. The view from a 45-degree angle shows the assembly. The top viewing window and the counter electrode port can be seen. There is also a port for the reference electrode out of view. **b)** The middle part, viewed from the bottom, shows how the electrical contact between working ITO electrode (for out-of-plane configuration) or titanium electrodes (for in-plane configuration) are isolated from the fluid in the middle chamber via an O-ring around the center bottom excitation/emission window. The fluid chamber also houses the reference and counter electrodes **c)** A cross-section of the assembled three parts, viewed from the side, illustrates how the chamber facilitates a high-resolution imaging scheme on an inverted microscope, as the objective lens makes contact with the coverslip from below via oil. Excitation and emission are collected through the same objective.

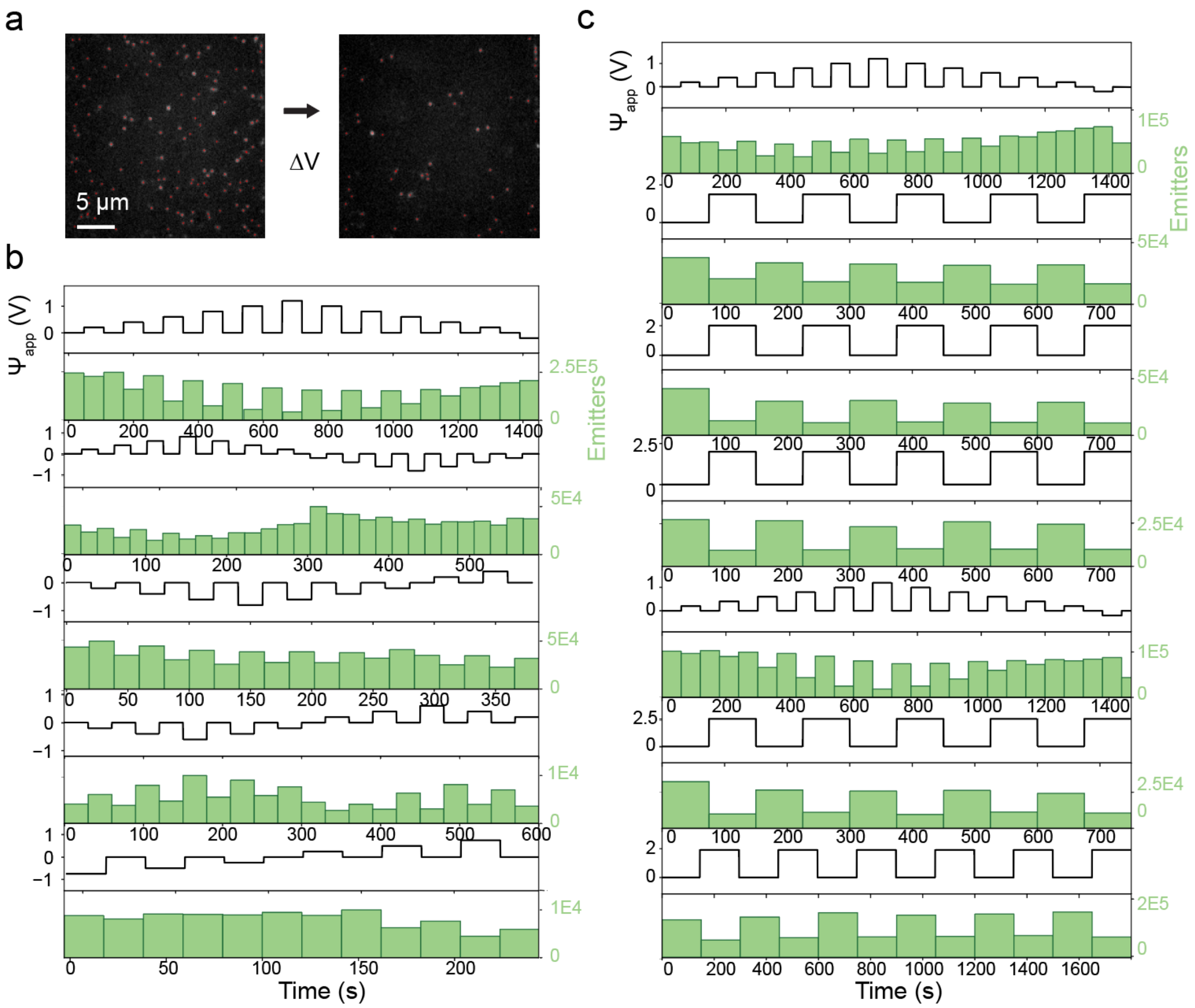

Supplementary Figure 2: Quantification of electrochemical modulation in methanol and acetonitrile. **a)** Single molecule emitters are tracked and counted by localizing using ThunderSTORM, (see *Materials and Methods*). Two example frames taken with 50 ms acquisition time during a cycling experiment show these localizations marked in red. Square pulses of various patterns were applied to different flakes in **b)** methanol and **c)** acetonitrile. The waveform is shown in black, and the corresponding binned localizations are shown in light green, below. Average changes from the same experiment's Ψ_{app} = 0 V counts were used to characterize the response of methanol-hBN emitters to potential. All potentials are reported against Ag/AgCl.

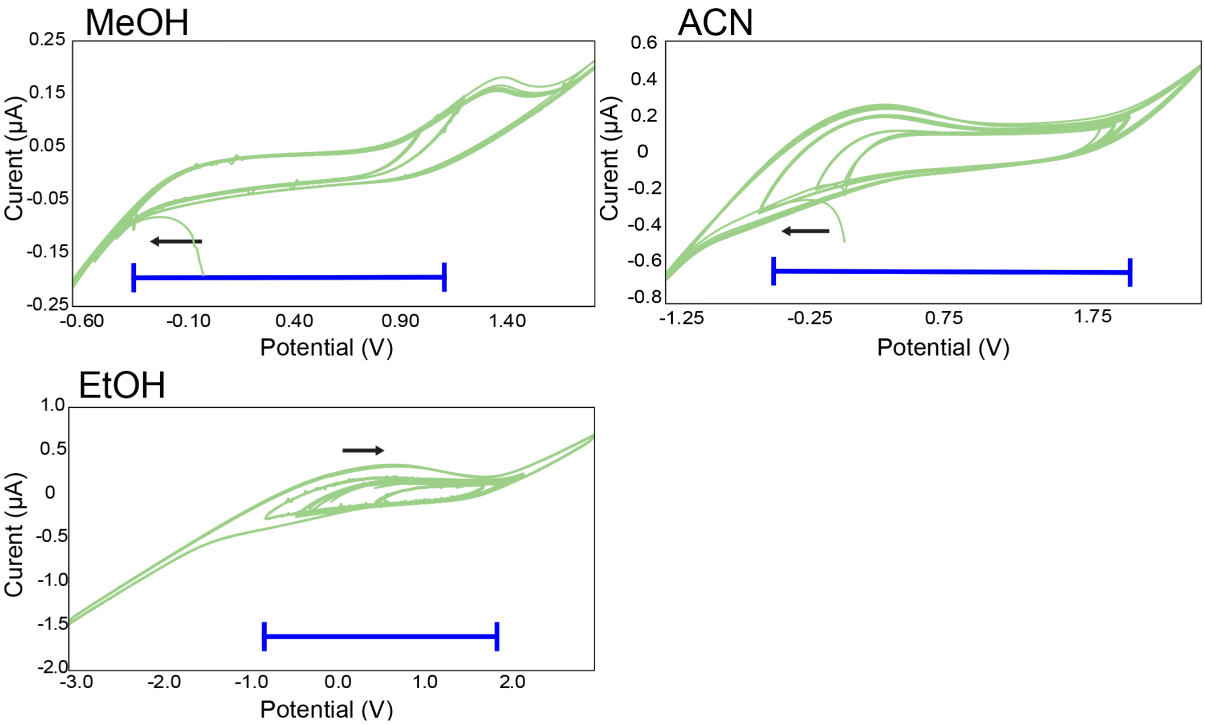

Supplementary Figure 3: Characterizing inert regions of solvents and ITO via cyclic voltammetry. Cyclic voltammetry was performed to determine the working potential range of solvents with an ITO working electrode vs Ag/AgCl leakless reference electrode in the three main solvents used in our experiments (methanol, acetonitrile, and ethanol). The stable working range is the flat region of the CV[1], shown by the blue bar and indicates the region wherein neither solvent nor electrode reacts. The scan rates were, 0.015 $V*s^{-1}$, 0.05 $V*s^{-1}$and 0.05 $V*s^{-1}$ for methanol, acetonitrile, and ethanol, respectively. The ITO coverslips have a diameter of 25 mm but only the center 8 mm diameter was exposed to solvent, corresponding to an active electrode surface area of 50 mm^2. The solvents were not used to quantify effects on hBN emitters with ITO outside these working regions.

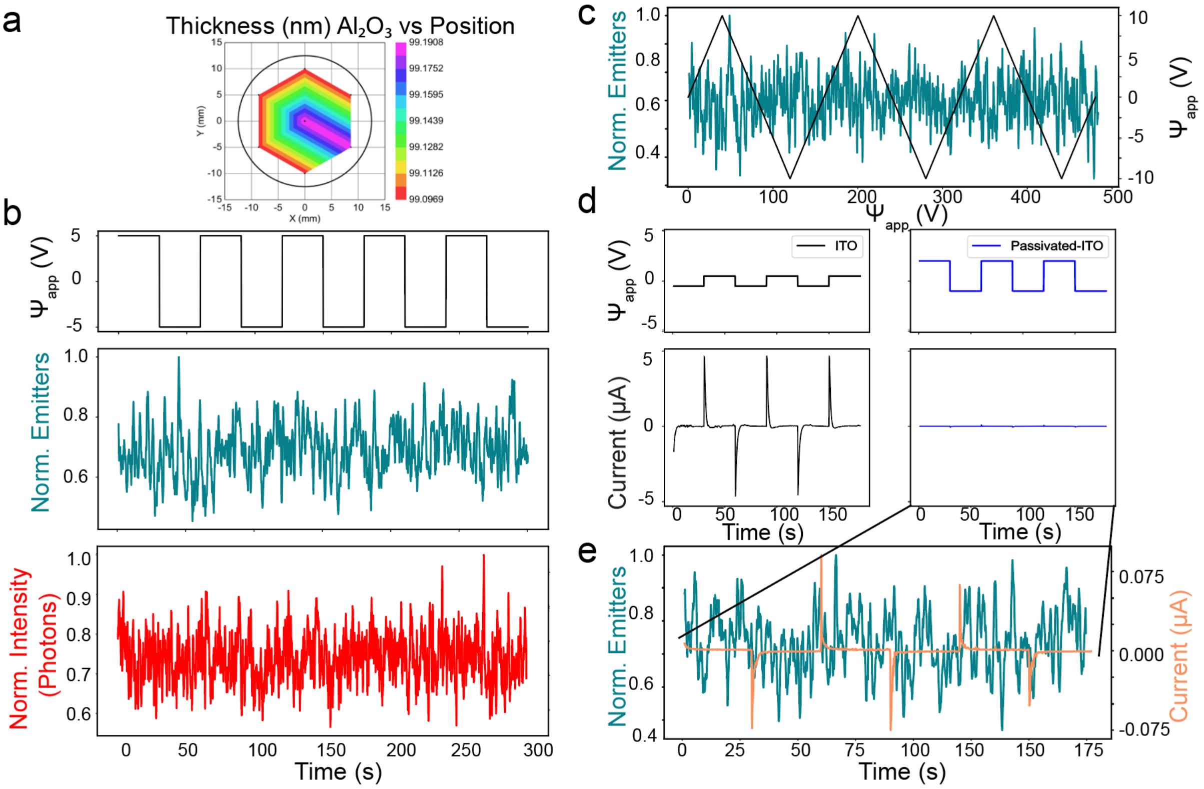

Supplementary Figure 4: Alumina oxide passivation. a) Ellipsometry confirms the 100 nm thick coating of alumina oxide deposited by atomic layer deposition over the ITO coverslip. **b)** In the absence of an active redox surface, the effect of cycling the voltage on emitter response was removed. A square voltage waveform applied to passivated ITO in methanol made no significant impact on the number of emitters on a flake (teal), even at high potentials (-/+5 V vs Ag/AgCl). There is also no modulation in average emitter intensity (red) per frame. **c)** A triangular waveform applied to a separate sample also shows no modulation of emitter counts even at very high potentials (-/+ 10 V, scan rate 250 mV/second). **d)** The un-passivated ITO currents (-/+0.5 V, black) compared to passivated ITO (-1 to +2 V, blue) in methanol are plotted on the same scale below the corresponding pulses. The magnitude of the passivated current is two orders of magnitude less than the un-passivated current, even for higher applied potentials. This indicates that, apart from minor currents, likely resulting from ion leakage through pores in Al_2O_3, the presence of the passivating layer effectively suppresses most reactions. This suppression seems to extend to our analyte reaction, as the effect of voltage cycling is effectively eliminated. **e)** The emitters are not modulated by the -1 to +2 V cycling (corresponding to the current trace above). The current from the bottom right panel of part d is scaled and can be seen to be less than 0.1 microamperes.

Supplementary Discussion on Stray-Fields

To evidence that the source of optical modulation was an active redox surface rather than an orientation/electric field effect, we introduced a "stray-field" configuration wherein the ITO thin film electrode was replaced by a glass slide patterned with a thin titanium electrode and flakes were placed adjacent to the electrodes at different distances. Modulation was consistent with the out-of-plane measurements, despite the change in electrode substrate and orientation of the field. The strength of the signal was highly dependent on proximity of flake to electrode **(Supplementary Figure 5)**, indicating we had a spatially sensitive electrochemical sensor. Compared to the flake placed at 13 micrometers from the working electrode, the emitter signal modulation of the flake placed at 30 micrometers is considerably smaller. This characteristic indicates that the analyte species quenching emitters must diffuse from the electrode. However, the persistence of the effect at a distance of 30 microns also indicates that the bulk concentration of this analyte is small enough to be altered by the alternating currents.

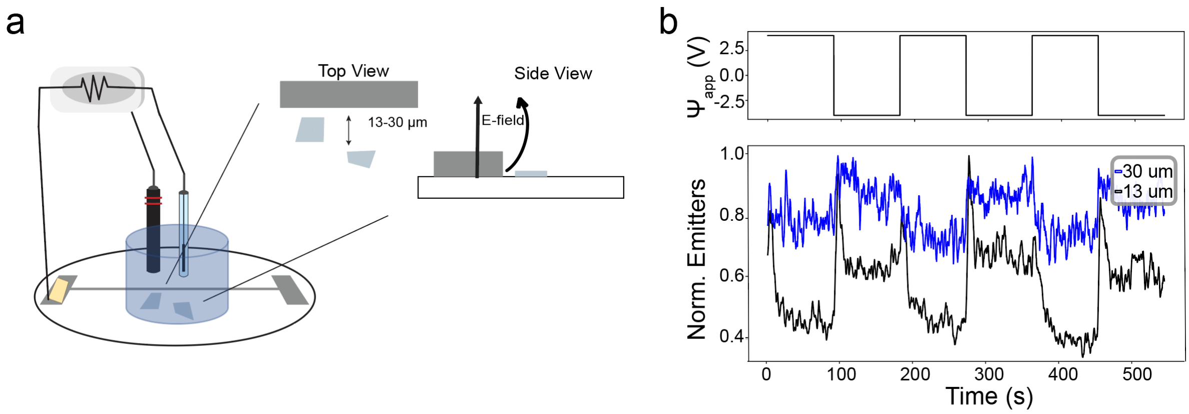

Supplementary Figure 5: Stray-field measurements demonstrate distance-dependent modulation strength. a) Schematic of the three-electrode configuration used to vary working electrode distance from flakes and apply stray electric field. Flakes were placed at a distance of 13 and 30 microns from the titanium working electrode. Glassy carbon and leakless Ag/AgCl were used as counter and reference electrodes, respectively which were connected to the potentiostat. **b)** The response of each flake to the same pulsed potential was monitored in acetonitrile. Although both flakes show the same behavior of decreased localizations at positive potentials, the magnitude is higher for the flake at 13 micrometers (black) than for the flake at 30 micrometers (blue) from the working electrode.

Supplementary Discussion on Kinetics

As discussed in the main text, the rate of reaction at the electrode is dependent on the electrochemical potential applied at the working electrode, k = k(V). The consistent and reversible modulation of analyte concentration is characterized in both reaction directions by exponential decay, indicative of a first order reaction. Although the optically-monitored reaction is between two reactants, the emitter and quencher, its rate depends primarily on the quencher concentration, as indicated by the direct dependency of rate on potential. This demonstrates a pseudo-first order reaction when the emitters are present at their equilibrium/excess concentration. However, to evaluate the reaction order, emitter traces were fit to functions indicative of first (linear $Log[Analyte]\ vs\ Time$) and second ($[Analyte]^{-1}\ vs\ Time$) order reactions. In the context of simple linear regression, the coefficient of determination (R^2) evaluates how much of the variability of the y variable can be explained by variation in x. Here we have high fluctuations of emitter counts (even at Ψ_{app} = 0 V), which explains the relatively low coefficients of determination. However, this does not impact our use of it to compare different models on the same data set. We also evaluated fits based on error and distributions of residuals over time (scatter plot) and residual distribution around 0 (histogram).

Based on our analysis, fitting to a first order reaction results in a higher goodness of fit and more evenly distributed residual values for the majority of potentials. **Supplementary Figure 6** shows an example pulse Ψ_{app} = 1.75 V but this was done for all potentials used in the kinetic analysis in **Figure 3**. It should be noted that calculating the kinetic constant for each electrochemical potential based on a second order reaction does result in the same trend as first order reaction fitting but errors are higher and the Tafel slope differs more from the current-derived value.

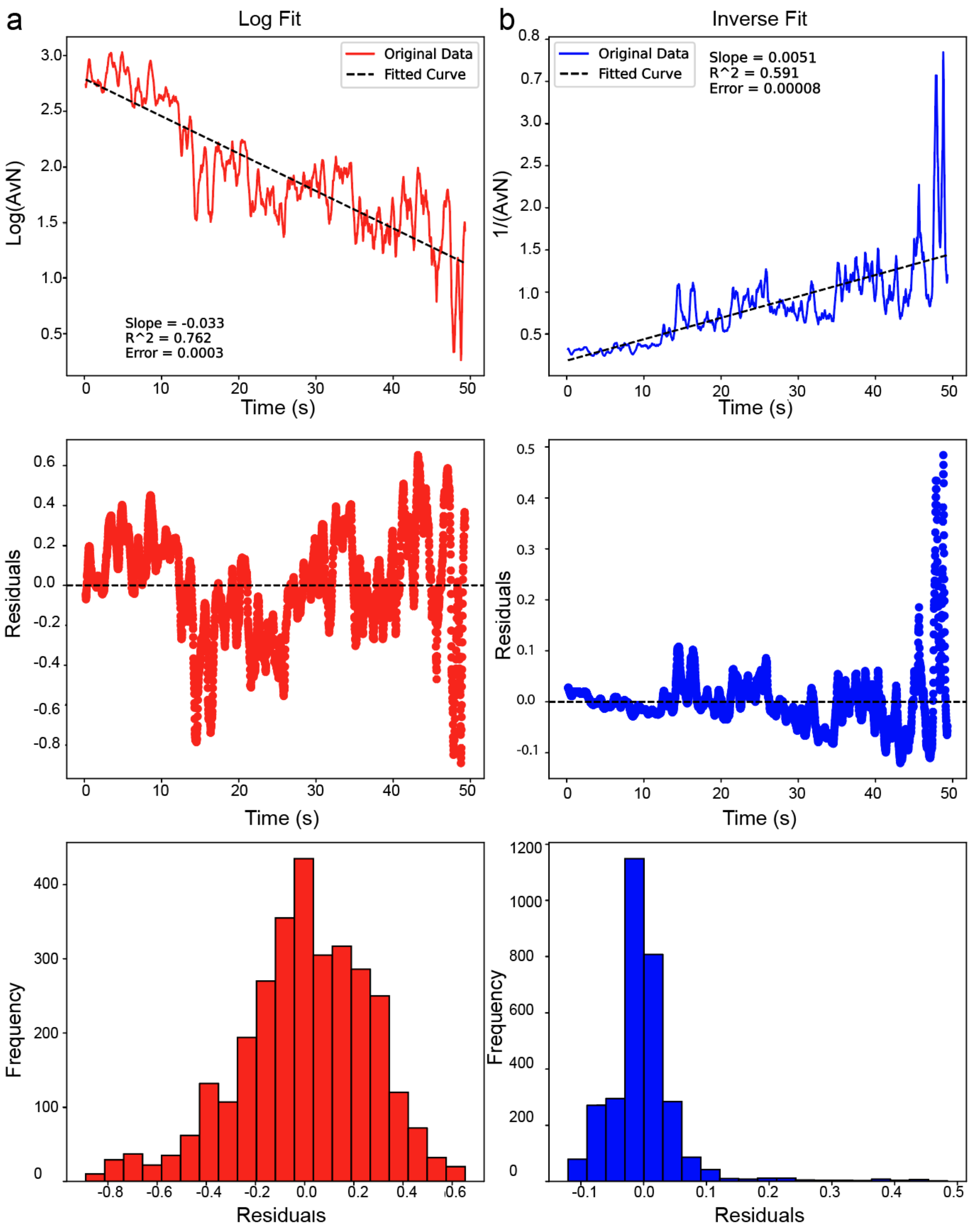

Supplementary Figure 6: Comparison of first order and second order fitting for emitter count decays in response to positive potentials. a) A first order reaction should have a linear $Log[Analyte]\ vs\ Time$ graph. Using the emitters as an optical readout for the quenching analyte, a linear fit was performed on $Log[Emitter]\ vs\ Time$ and was evaluated by R^2 and residual distributions. The example here shows emitter behavior for Ψ_{app} = 1.75 V. **b)** Alternatively, a second order reaction should have a linear $[Analyte]^{-1}\ vs\ Time$ graph. Thus, a linear fit was also performed on $[Analyte]^{-1}\ vs\ Time$ and was evaluated by R^2 and residual distributions.

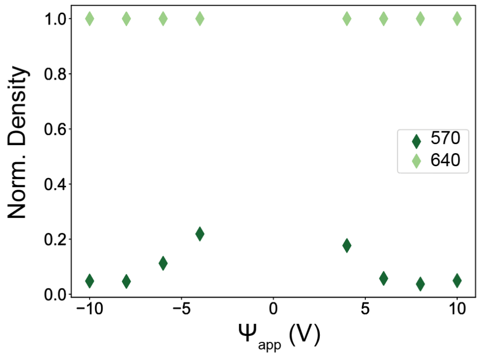

Supplementary Figure 7: In-Plane spectral analysis. At high electrochemical potentials the relative density of the secondary group of emitters (570 nm peak) is particularly reduced while the main peak around 640 nm is consistently the most prominent. This agrees with our findings in **Figure 3d-f**. Here we use titanium electrodes rather than ITO and the electrochemical potentials probed are higher in magnitude, but the surface is smaller.

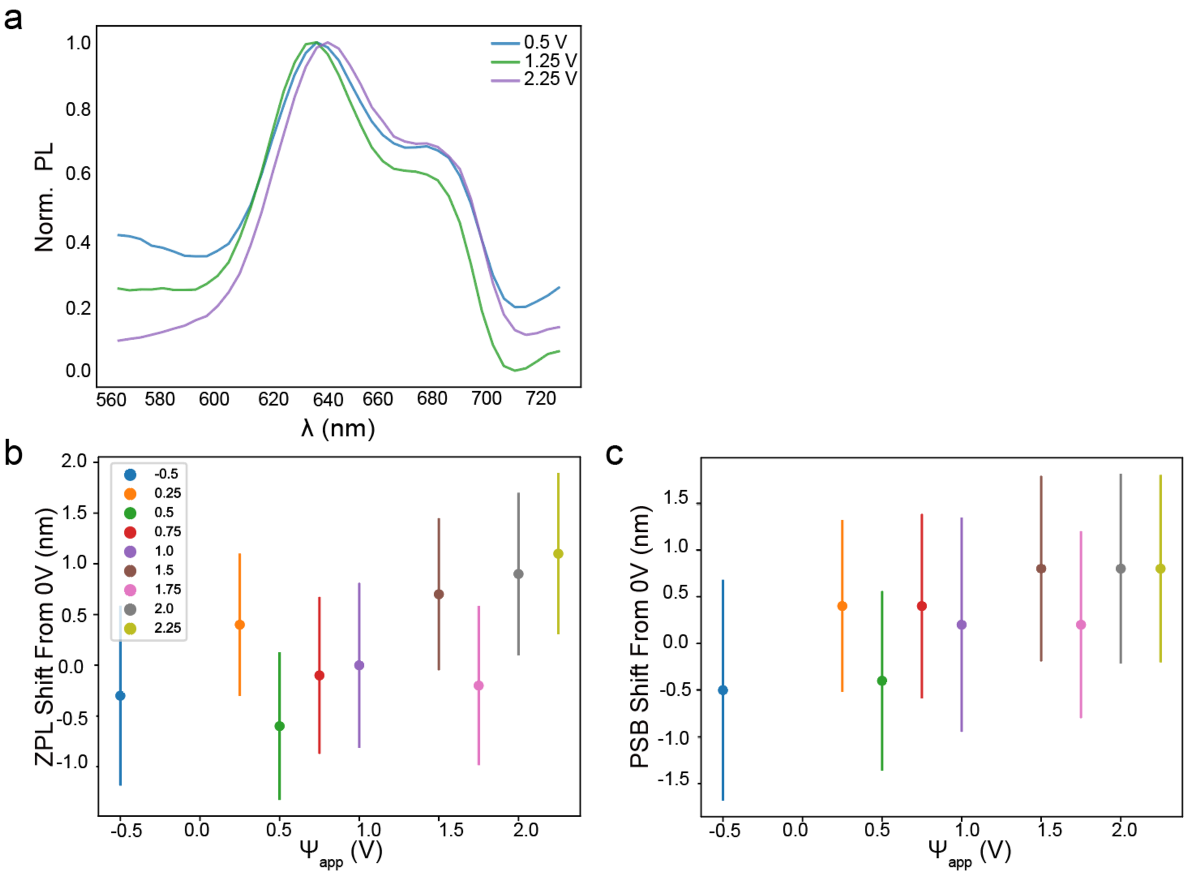

Supplementary Figure 8: Out-of-plane averaged spectra shows no significant stark shift. a) The ensemble-averaged spectrum of emitters measured at three different potentials in acetonitrile is presented. The averaging process differs from the use of individual emitter spectra, as depicted in **Figure 3d**, shown as a histogram and KDE. **b)** When fitting the averaged spectra to two Lorentzians (as described in *Materials and Methods*) the ZPL is obtained. Plotting ZPL shift from Ψ_{app}= 0 V for

increasing potentials revealed no discernible trend. **c)** The PSB shift from 0 V also showed no significant trend as the electrochemical potential increased.

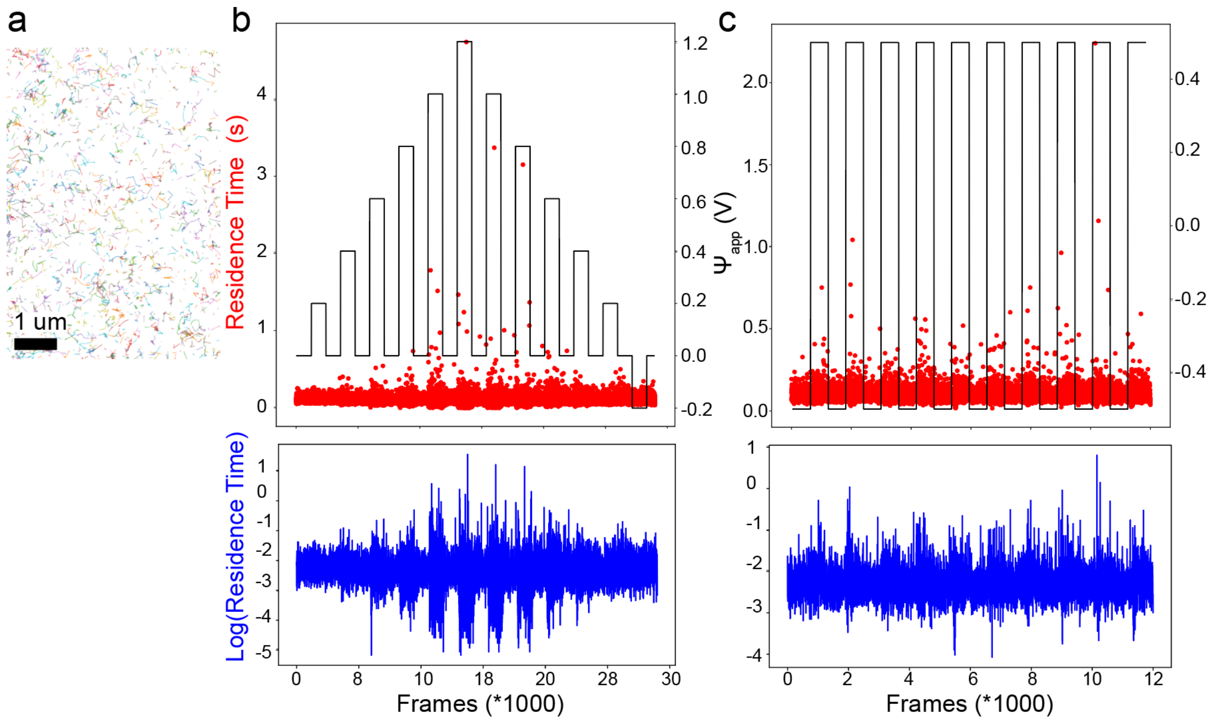

Supplementary Figure 9: Residence time analysis. a) By linking sub-diffraction localizations (ThunderSTORM) via single molecule tracking techniques (Trackpy Python implementation of Crocker-Grier algorithm) we determined the residence times of emitters. We defined the residence time as the number of subsequent frames an emitter is present from that frame forward. We characterize residence times by single trajectories, not single sites. Following a rationale detailed in previous work[2], we used a search range of 345 nm, adapted to our exposure time of 50 ms. Methanol-hBN surface emitter tracks at 0 V vs Ag/AgCl are shown after accumulating over 500 frames (50 ms per frame). **b)** A square waveform voltage ramp in methanol shows the changing distribution of residence times at increasingly positive potentials. The top panel just shows the average residence time for all emitters in that frame while the bottom panel shows the log of that average. The shape indicates that at positive voltages certain emitters are reduced from the beginning, which is intuitive with counts analysis in main text **Figures 1 and 2**. However, there is also an unexpected broadening of residence distribution, emitters that persist through the positive potentials seem to persist longer. **c)** The residence times (red) and log of residence times (blue) is also displayed for Ψ_{app}= +/-0.5 V in methanol and demonstrates the same trend.

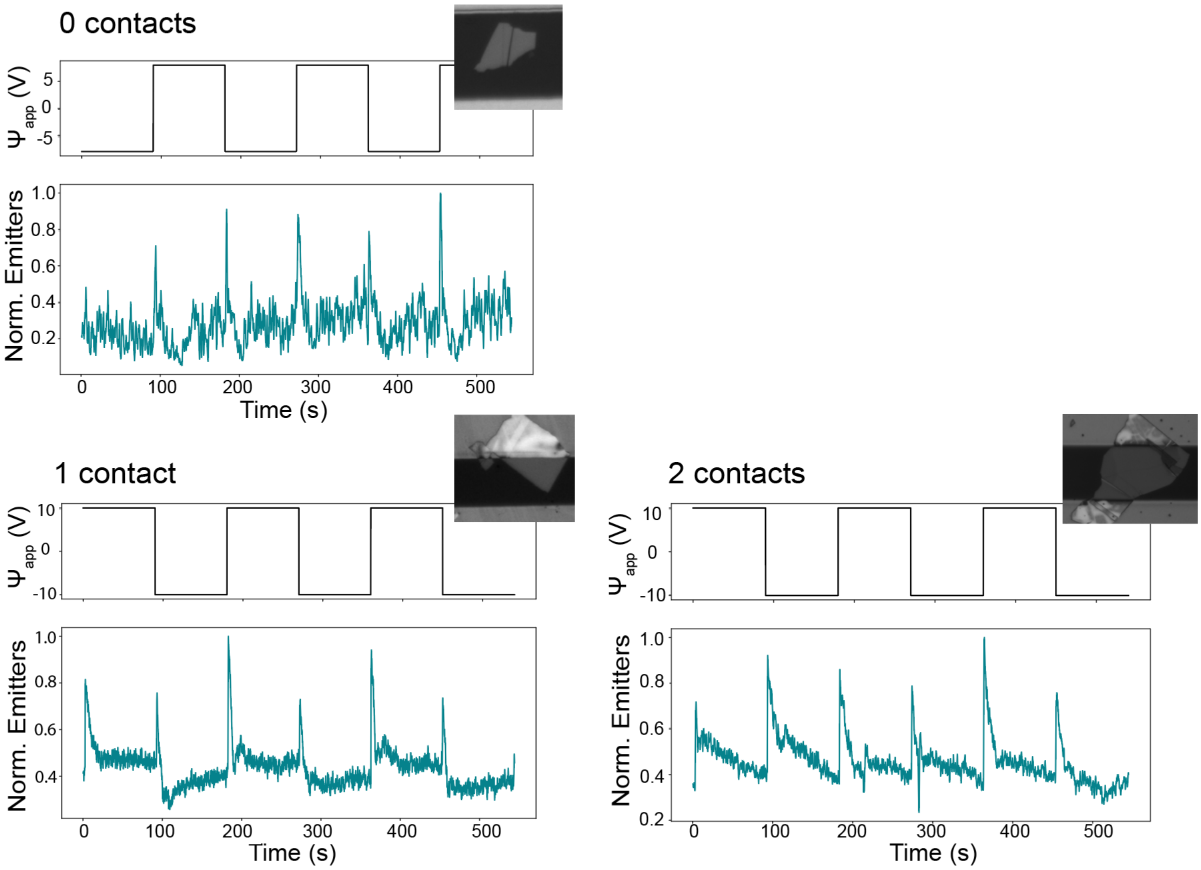

Supplementary Figure 10: Contact configuration analysis. We conducted experiments with various contact configurations to rule out the possibility of emitter modulation being caused by charge injection from the electrodes to hBN. Despite hBN being an insulator, we considered the possibility that high electrochemical potentials could impact defect charge states. Flakes were tested with no contact to either electrode, contact to one electrode, and contact to both electrodes. However, all configurations exhibited the same overall behavior. The variance in signal strength of these capacitive-looking switches can be understood as being due to larger distances between the electrode surface and the flake in the configurations with 0 contacts and 1 contact.

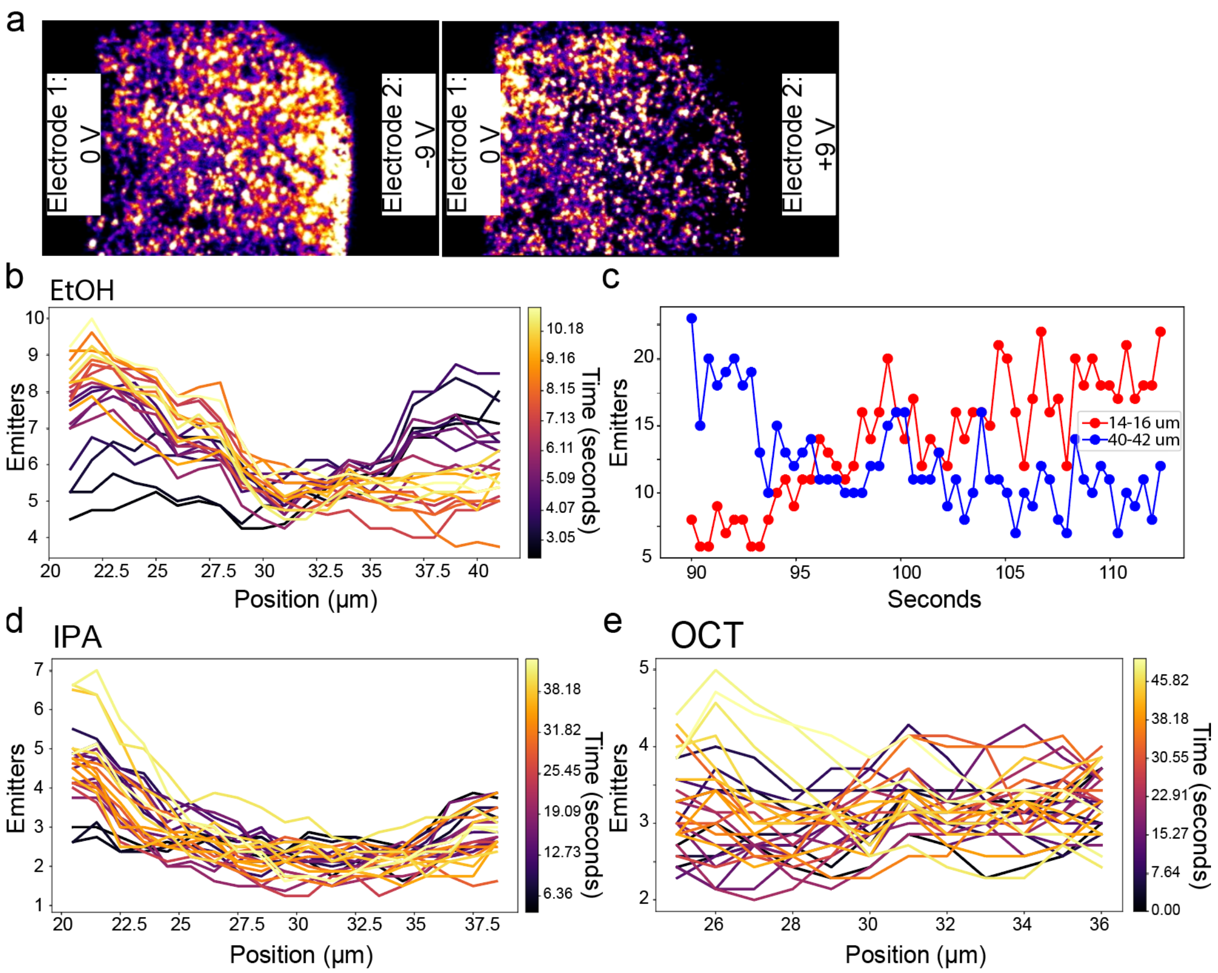

Supplementary Figure 11: Monitoring concentration gradients in time. a) Two screenshots from the experiment in **Figure 4** and **Supplementary Video 2** demonstrating the concentration gradient coinciding with electrode polarization switch. **b)** The concentration gradient can be seen increasing over time after the switch in electrode polarization in the in-plane configuration with ethanol (EtOH). Switching the electrode polarization shows the switch in concentration gradient from high emitters on the right at the beginning to high on the left after 7 seconds. The time is reported from the time of the switch in electrode polarization. **c)** The change in local concentration for the same experiment in EtOH is also visualized by plotting the number of emitters in a 2 μm distance from the top electrode (red) and bottom electrode (blue). **d)** In isopropyl alcohol (IPA) we can also effectively monitor the concentration gradient buildup as the voltage is applied in the first cycle (not switching of polarization as in part a). The gradient takes about 3 times as long to build. **e)** Finally, in octanol (OCT), similar data is presented but the change in emitter density is considerably less, even after 45 seconds. Acetonitrile is shown in the main text (**Figure 4e**).

Supplementary Discussion on Mechanisms

Identifying and Excluding Candidate Reactant Species

Although we worked in the inert range of the electrodes and solvent (**Supplementary Figure 3**), products of an oxidation reaction of the organic solvent itself (including acetaldehyde, carboxylic acids, and CO_2) were considered as possible contributors to altered fluorescence. Intermediates and products of the bulk solvent were ultimately ruled out because the onset potential of the solvent reactions (indicated by curvature in the CVs) did not appear to coincide with the onset of our emitter modulation by electrochemical potential. Oxidation potentials depend heavily on the reaction conditions, such as pH, electrolyte, electrode type, etc., so it can be problematic to compare onset potentials between studies, especially since we were working in organic solvents without supporting electrolyte. Therefore, to understand the onset oxidation of a bulk solvent reaction in our system we generally refer to our CVs (**Supplementary Figure 3**). It has previously been shown that the onset of methanol oxidation with non-noble metals is generally >=1.2 V vs RHE[3] (>=1.397 V vs Ag/AgCl) and a current peak around this onset potential can be seen in our methanol CV. Thus, if the modulating species was an intermediate or product of this reaction, then the effect should begin around this point but the effect on emitters is seen at potentials much lower, even below 0.5 V vs Ag/AgCl in methanol. Furthermore, in ethanol the onset potential as seen from the CV is around 2 V vs Ag/AgCl, yet the effect is clearly seen on emitters at Ψ_{app}= +1 V (**Supplementary Figure 12**). Finally, since the current trace's curvature cannot be related to the effect on emitters, we are likely optically monitoring a reaction that is a subset to the overall current reported by the potentiostat.

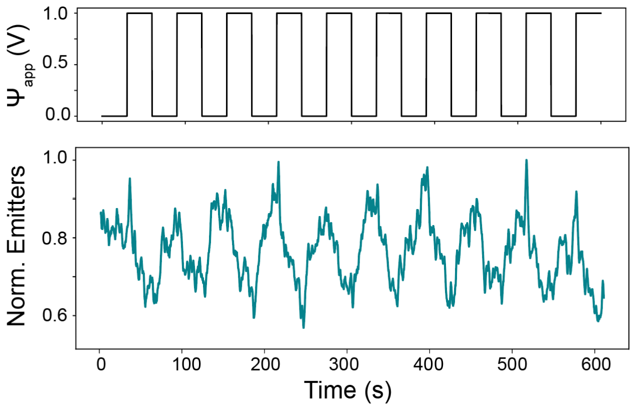

Supplementary Figure 12: Ethanol cycling. A square waveform was applied to the ITO electrode in ethanol in the three-electrode out-of-plane configuration to show the consistent behavior of emitters in other solvents. At +1 V the number of emitters decreases compared to 0V vs Ag/AgCl.

After eliminating bulk solvent reactions as the source of modulation, we sought to understand the activity at the electrode surface by examining the presence of trace species within the system and how their concentrations would respond to changes in electrochemical potential. For example, dioxygen would be present in the system from contact with the environment and its solubility in organic solvents[4]. This dioxygen could then be electrochemically reduced to oxygen radicals, present in trace amounts, which is a well-known quencher of fluorescent emitters[5]. However, this mechanism is inconsistent in that oxygen radicals are more likely to form at negative potentials while emitters in

our system are instead quenched at positive potentials. Moreover, the extent of reaction is indicated by the extent of the change in center of mass, delta y, which relates directly to the solubility of water in the solvent (**Supplementary Figure 11**). Oxygen solubility increases with increasing chain length of the alcohols, but the gradient build-up is far lower in the long-chained octanol, indicating that oxygen is not the modulating species.

Other radicals were also considered, such as products of an oxidation reaction of the organic solvent: for example, alkoxy radicals may be formed at the electrode surface during oxidative cycling in alcohols but these radicals are highly reactive and would have short lifetimes (microseconds)[6], making it unlikely they would influence an electrode 30 μm away, such as is seen in the stray-field measurement (**Supplementary Figure 5**). Considering a diffusion constant on the order of magnitude of 10^{-9} m^2/s and assuming linear diffusion, diffusion of the radical from electrode to flake would take ~0.5 seconds, 5 orders of magnitude larger than the average lifetime of the alkoxy radicals.

Experiments Showing the Involvement of Water and H^+

Another trace species which would be present in all the solvents is water. Water and protons are known quenchers of red emitting dyes[5,7,8] and water would be present initially in the as-purchased solvents (100-200 ppm for acetonitrile and methanol and over 1000 ppm for ethanol)[9]. Water concentration would also be somewhat higher than as-purchased due to the exposure of the solvent to the environment–the cell was not air-tightly sealed during any experiment until anhydrous experiments (discussed in the following) and was not prepared in a glovebox.

To test the hypothesis that hBN emitters are quenched by interaction with protons, the concentration of which can be modulated by water oxidation, we deliberately introduced water **(Supplementary Figure 13)**. As a result of introducing 3 M water into methanol, emitters were quenched by more than 50% and quenching percent was positively correlated with increasing concentration of water. We then directly introduced the proposed quencher, H^+ (in the form of HCl), to methanol. This resulted in the same quenching trend as water but to a higher extent, reaching more than 50% quenching at only 70 mM, confirming that quenching was positively correlated with water and particularly H^+.

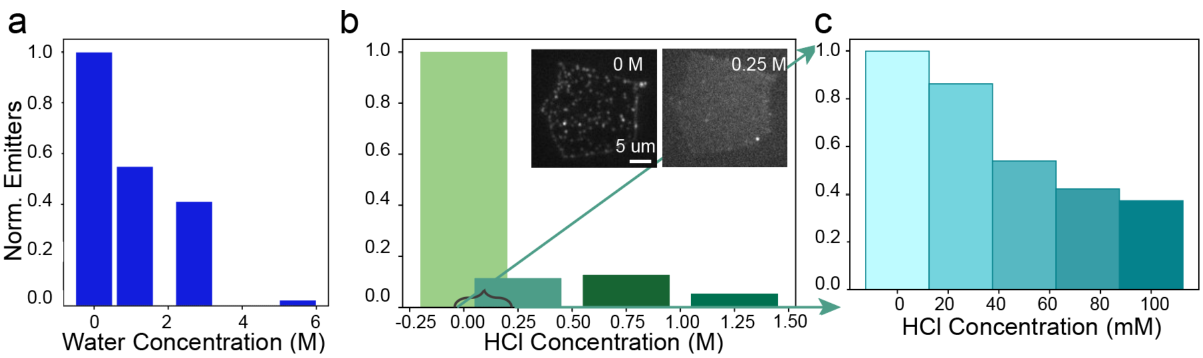

Supplementary Figure 13: Deliberate introduction of quenchers. a) The average number of emitters per frame for a single flake for 2400 frames is plotted against increasing water concentration. Emitters per frame is normalized. As water concentration increases the concentration of emitters in methanol decreases. At 2.5 M the emitter counts are decreased by more than 60%. **b)** The average number of emitters per frame is plotted in for increasing concentration of hydrochloric acid. The concentration was increased by mixing pure methanol with a stock of 5 M HCl in methanol, in which the HCl had

been bubbled in. The inset images show the flake imaged in pure methanol vs with 250 mM HCl. **c)** A lower concentration range was tested using a fresh sample. This showed that emission is quenched by more than 50% at only 70 mM.

We then considered that removing the reactant candidate (water) should eliminate the modulation of the quencher concentration. To remove adventitious water, we used an activated 3Å molecular sieve over 48 hours in a glovebox to dry acetonitrile. The water content in acetonitrile after this treatment should be imperceptible[9]. The acetonitrile was then loaded in ambient conditions into an electrochemical chamber. The electrochemical chamber was sealed from the environment and cycling experiments were performed and their results, referred to as "dehydrated" were compared to the results from normal acetonitrile, referred to as "wet". Consistent with our proposal, this drying removed the effect of voltage cycling when switching between (-/+0.5 V) whereas wet acetonitrile has a strong response to this pulse (**Supplementary Figure 14**). When the magnitude of the pulses increased, an effect on emitter counts was seen even in the dry acetonitrile but still to a lesser extent, indicating that water plays an important role in our modulating reaction mechanism. One should note that, due to technical limitations, the experiment (including the chamber assembly) was not performed in a glovebox, so the introduction of water by contact with the environment was possible during this time.

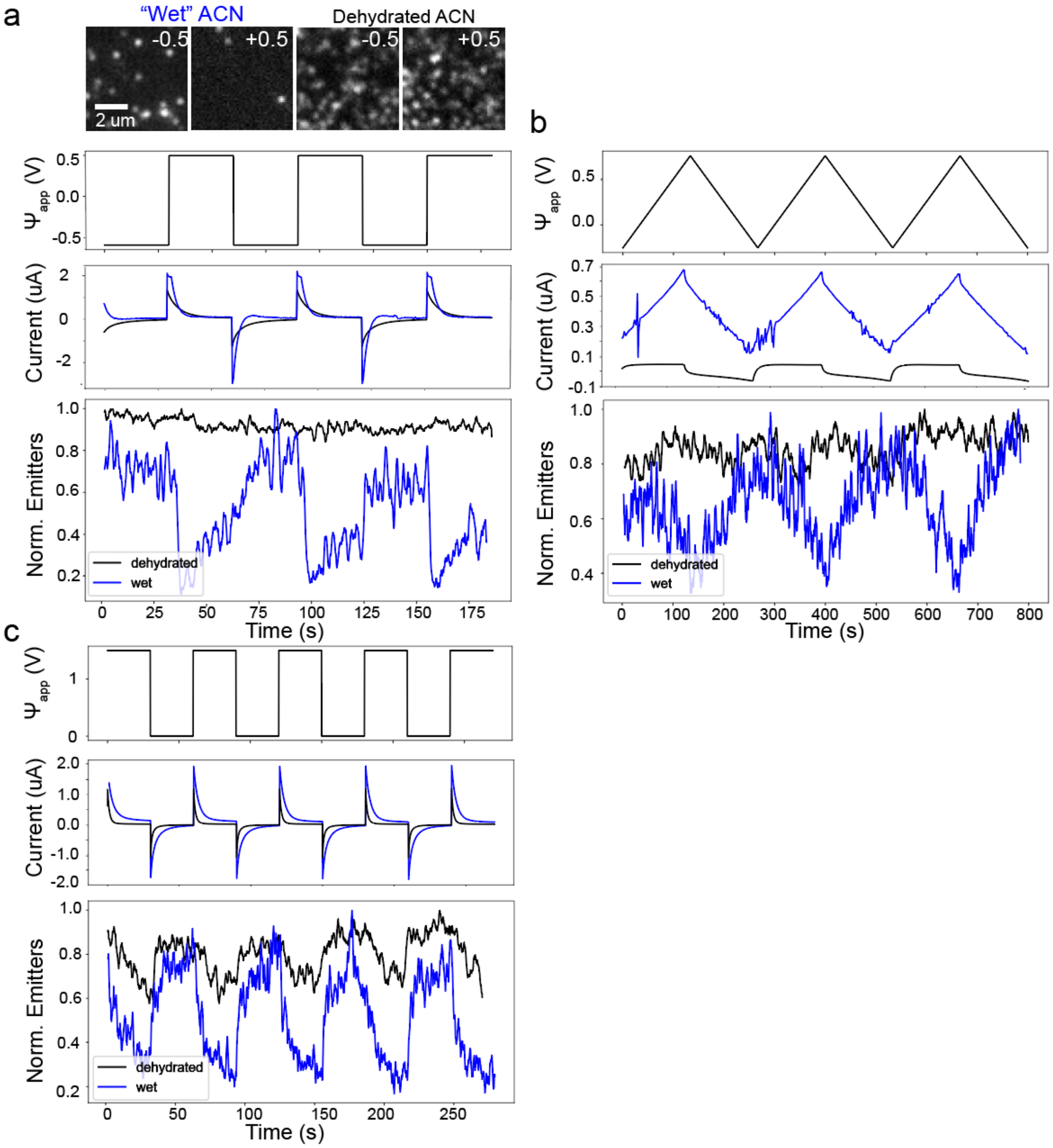

Supplementary Figure 14: Deliberate Removal of Water. **a)** Example frames show the visible difference in modulation extent between wet and dehydrated acetonitrile when cycling between Ψ_{app}= -/+0.5 V at the ITO electrode in the three-electrode out-of-plane configuration. The corresponding applied waveform, currents, and normalized emitters per frame are shown below. Wet acetonitrile is shown in blue while dehydrated acetonitrile is shown in black. Dehydration effectively eliminates modulation by cycling and decreases the current. **b)** A triangular waveform was applied to the ITO working electrode in the dehydrated acetonitrile and the currents and normalized emitters per frame are shown in comparison to the normal, wet, acetonitrile. Modulation of emitters by electrochemical potential is again effectively eliminated. **c)** Finally, the effect of higher applied electrochemical potential (Ψ_{app} = + 1.25) recovers the impact on dry acetonitrile although the effect is still lower than in wet acetonitrile. It should also be noted that the experiments were done in the order of presentation and any leakage of humidity through our sealing would occur over time.

From the modulations of water and H^+ concentrations in our solution we can confirm that these species play an important role in emitter quenching. We thus propose that the emitter density is modulated by the electrochemical reduction/oxidation of water which results in changing concentration of H^+. In our in-plane experiments, oxidation and reduction of H^+ can occur in the same field of view, explaining the increase of emitters on one side and decrease on the other. This is schematically illustrated in **Supplementary Figure 15**.

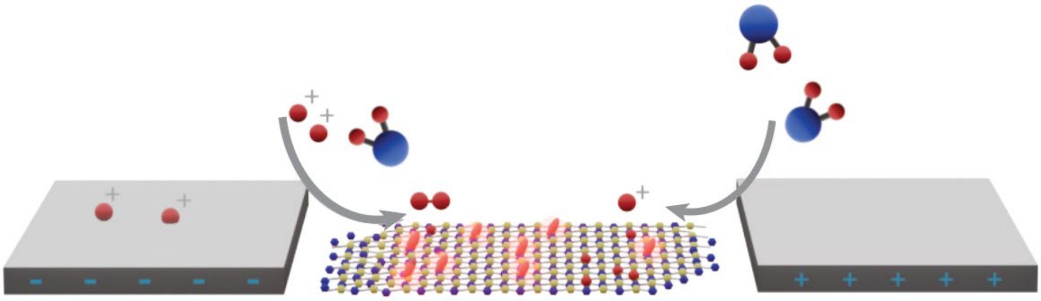

Supplementary Figure 15: Interpretation of modulation mechanism. An interpretation of the system that was shown optically in **Figure 4**. In this interpretation the modulation is due to the reduction/oxidation of H^+ and water, respectively. H^+ can be catalytically reduced to H_2 at the surface of the titanium electrode or recombined into water using molecular oxygen. Alternatively, at the positive electrode, water is oxidized to form H^+, which can quench emitters. The emitters then appear at higher density near the negative electrode whereas diminished fluorescence is observed near the positive electrode.

Proposed Electrochemical Mechanism

We propose that the modulation of emitters during electrochemical cycling results from the reduction/oxidation reactions of water, which changes the concentration of H^+, ultimately quenching the emitters. The oxidation reaction at positive potentials is described by electrolysis, the electrochemical splitting of liquid water into hydrogen and oxygen using electricity[10]. Meanwhile, at the cathode, higher emitter density is recorded as H^+ is consumed (e.g. reduced to H_2 or recombined into water). One should note that the transient behavior of emitters is not induced by adding water or introducing electrochemical cycling but is present in all experiments on hBN organic solvent emitters, as previously reported[2].

As was discussed briefly in the main text, the increased reactivity of water in acetonitrile can be understood by the alteration of its molecular aggregation behavior, with its oxidation potential shown to steadily decrease with decreasing water concentrations[11] . However, just as our redox modulation is not unique to acetonitrile, the isolation of water molecules in mixtures has also been observed and modeled with ionic liquids/water[12] and methanol/water[13], respectively. Moreover, in our experiments we have the addition of irradiating light, which has been seen to increase reactivity and drive the water splitting reaction[14,15].

Finally, the development of H^+ sensors based on fluorescence is valuable to the innovation of *in-operando* probes. In our system, hBN organic solvent emitters were shown to be highly sensitive to H^+ concentration in methanol, the monitoring of which is decidedly relevant to development of methanol fuel cells[16,17]. The spatiotemporal resolution of our system also means that the H^+ concentration could be monitored as a function of distance from electrode or membrane over time.

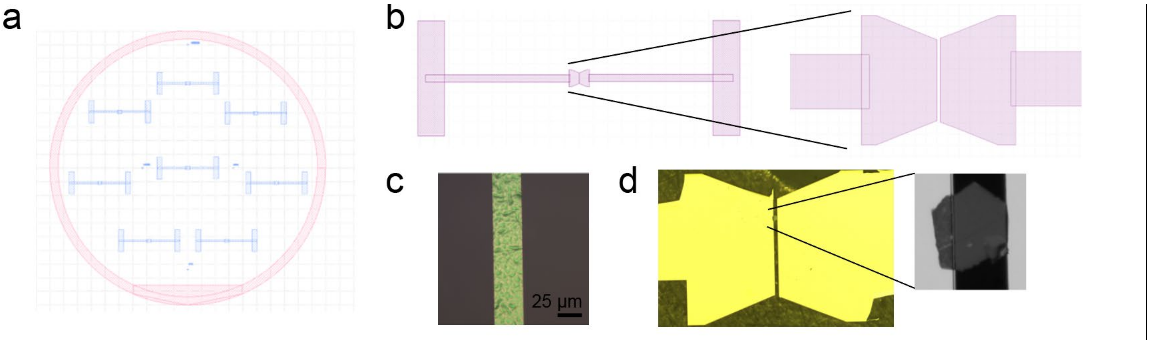

Supplementary Figure 16: Wafer for in-plane electrode fabrication. **a)** In-plane and stray-field electrodes are patterned onto 8 coverslips at a time using E-beam evaporation with a Si_3N_4 shadow mask. **b)** The stencil mask was created using direct laser writing and positive photolithography. Thus, everywhere shown in pink was etched away and a thin nitride membrane remains. The distance between electrodes was varied by controlling the width of the remaining nitride membrane. **c)** The optical microscope image shows an example nitride membrane that is 30 µm wide. **d)** The result of evaporating 100-250 nm of titanium onto a 25 mm diameter glass coverslip using the stencil mask is seen. A flake has been transferred deterministically between the electrodes using a PDMS stamp.

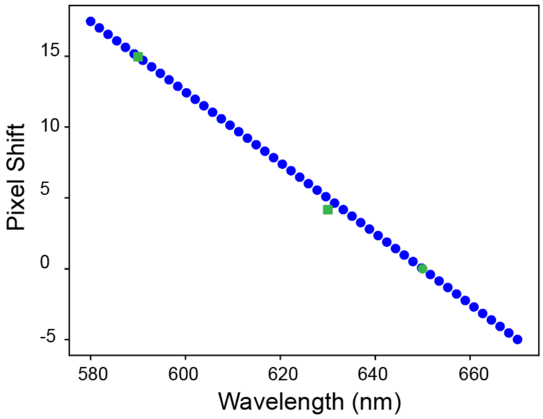

Supplementary Figure 17: Bead calibration of spectral SMLM. Linear calibration for the vertical shift in the spectral channel as a function of emission wavelength was done using broadband beads and narrow bandpass filters. On the spectral path there is a dispersive prism that leads to an approximately linear shift in the position of the emitter relative to the wavelength. Recall from the *Materials and Methods* section that $(x_{SPEC}, y_{SPEC}) = A \times (x_{LOC}, y_{LOC}) + B$ where A is a 2×2 matrix and B is a column vector. The B vector was determined via this linear calibration. This sketch is of the same method previously reported[18] with updated calibration.

Captions For Supplementary Videos

Supplementary Video 1: Out-of-plane emitter control. The video included is a wide-field view of an hBN flake immersed in acetonitrile while an electrochemical potential of the ITO working electrode is cycled in the three-electrode out-of-plane configuration between +1.5 V and 0 V vs Ag/AgCl. The change in potential induces a change in density of emitters. The raw data response is shown in light teal while a rolling average of 20 frames is shown in dark teal. Current is shown in orange. This data corresponds to a flake used in spectral analysis (data presented in **Fig. 3c–e**). Continuous ~1.6 kW cm^{-2} illumination with a 561 nm laser is used. The original images were acquired at a rate of 50.091 ms per frame but here we combined frames to present a lighter video with a lower sampling rate (1 s). The total experiment took 750 seconds, but we present the image stack at a rate of 10 frames per second to make the video only 75 seconds. The scale bar is 5 µm.

Supplementary Video 2: In-plane emitter control. Here we present a wide-field video of the hBN flake shown in **Figure 4b-d** in between two titanium electrodes. Continuous ~1 kW cm^{-2} illumination is used with a 561 nm laser while the polarization of the electrodes is cycled in the two-electrode in-plane configuration, inducing a change in density of emitters correlated with potential. The high-density region follows the negatively charged electrode. The original images were acquired with a 50.091 ms rate, but here we combined frames to present a lighter video with a lower sampling rate (2.5 s). The total experiment took 540 seconds, but we display 5 of the stacked images per second, making make the video around 40 seconds. The scale bar is 5 µm.

Supplementary References

(1) Benck, J. D.; Pinaud, B. A.; Gorlin, Y.; Jaramillo, T. F. Substrate Selection for Fundamental Studies of Electrocatalysts and Photoelectrodes: Inert Potential Windows in Acidic, Neutral, and Basic Electrolyte. *PLOS ONE* **2014**, *9* (10), e107942. https://doi.org/10.1371/journal.pone.0107942.

(2) Ronceray, N.; You, Y.; Glushkov, E.; Lihter, M.; Rehl, B.; Chen, T.-H.; Nam, G.-H.; Borza, F.; Watanabe, K.; Taniguchi, T.; Roke, S.; Keerthi, A.; Comtet, J.; Radha, B.; Radenovic, A. Liquid-Activated Quantum Emission from Pristine Hexagonal Boron Nitride for Nanofluidic Sensing. *Nat. Mater.* **2023**, *22* (10), 1236–1242. https://doi.org/10.1038/s41563-023-01658-2.

(3) Wang, J.; Zhang, B.; Guo, W.; Wang, L.; Chen, J.; Pan, H.; Sun, W. Toward Electrocatalytic Methanol Oxidation Reaction: Longstanding Debates and Emerging Catalysts. *Adv. Mater.* **2023**, *35* (26), 2211099. https://doi.org/10.1002/adma.202211099.

(4) Ramesh, H.; Mayr, T.; Hobisch, M.; Borisov, S.; Klimant, I.; Krühne, U.; Woodley, J. M. Measurement of Oxygen Transfer from Air into Organic Solvents. *J. Chem. Technol. Biotechnol.* **2016**, *91* (3), 832–836. https://doi.org/10.1002/jctb.4862.

(5) Maillard, J.; Klehs, K.; Rumble, C.; Vauthey, E.; Heilemann, M.; Fürstenberg, A. Universal Quenching of Common Fluorescent Probes by Water and Alcohols. *Chem. Sci.* **2021**, *12* (4), 1352–1362. https://doi.org/10.1039/D0SC05431C.

(6) Pryor, W. A. Oxy-Radicals and Related Species: Their Formation, Lifetimes, and Reactions.

(7) Agmon, N. Elementary Steps in Excited-State Proton Transfer. *J. Phys. Chem. A* **2005**, *109* (1), 13–35. https://doi.org/10.1021/jp047465m.

(8) Yang, D.; Wu, X.-T.; Cao, X.-J.; Zhao, B.-X. A Reversible Ratiometric Fluorescence Probe for Fast Detection of Trace Water in Different Organic Solvents. *Dyes Pigments* **2019**, *170*, 107558. https://doi.org/10.1016/j.dyepig.2019.107558.

(9) Williams, D. B. G.; Lawton, M. Drying of Organic Solvents: Quantitative Evaluation of the Efficiency of Several Desiccants. *J. Org. Chem.* **2010**, *75* (24), 8351–8354. https://doi.org/10.1021/jo101589h.

(10) Dau, H.; Limberg, C.; Reier, T.; Risch, M.; Roggan, S.; Strasser, P. The Mechanism of Water Oxidation: From Electrolysis via Homogeneous to Biological Catalysis. *ChemCatChem* **2010**, *2* (7), 724–761. https://doi.org/10.1002/cctc.201000126.

(11) Hidalgo-Acosta, J. C.; Scanlon, M. D.; Méndez, M. A.; Peljo, P.; Opallo, M.; Girault, H. H. Enhanced Reactivity of Water Clusters towards Oxidation in Water/Acetonitrile Mixtures. *ChemElectroChem* **2016**, *3* (12), 2003–2007. https://doi.org/10.1002/celc.201600190.

(12) Zhao, C.; Bond, A. M. Photoinduced Oxidation of Water to Oxygen in the Ionic Liquid BMIMBF4 as the Counter Reaction in the Fabrication of Exceptionally Long Semiconducting Silver-Tetracyanoquinodimethane Nanowires. *J. Am. Chem. Soc.* **2009**, *131* (12), 4279–4287. https://doi.org/10.1021/ja806893t.

(13) Zhang, N.; Shen, Z.; Chen, C.; He, G.; Hao, C. Effect of Hydrogen Bonding on Self-Diffusion in Methanol/Water Liquid Mixtures: A Molecular Dynamics Simulation Study. *J. Mol. Liq.* **2015**, *203*, 90–97. https://doi.org/10.1016/j.molliq.2014.12.047.

(14) Sheridan, M. V.; McLachlan, J. R.; González-Moya, J. R.; Cortés-Medina, N. D.; Dares, C. J. Indium Tin-Doped Oxide (ITO) as a High Activity Water Oxidation Photoanode. *ACS Appl. Mater. Interfaces* **2021**, *13* (33), 40127–40133. https://doi.org/10.1021/acsami.1c11298.

(15) Abe, R.; Higashi, M.; Domen, K. Facile Fabrication of an Efficient Oxynitride TaON Photoanode for Overall Water Splitting into H2 and O2 under Visible Light Irradiation. *J. Am. Chem. Soc.* **2010**, *132* (34), 11828–11829. https://doi.org/10.1021/ja1016552.

(16) Zhao, H.; Shen, J.; Zhang, J.; Wang, H.; Wilkinson, D. P.; Gu, C. E. Liquid Methanol Concentration Sensors for Direct Methanol Fuel Cells. *J. Power Sources* **2006**, *159* (1), 626–636. https://doi.org/10.1016/j.jpowsour.2005.09.067.

(17) Joghee, P.; Malik, J. N.; Pylypenko, S.; O'Hayre, R. A Review on Direct Methanol Fuel Cells – In the Perspective of Energy and Sustainability. *MRS Energy Sustain.* **2015**, *2*, E3. https://doi.org/10.1557/mre.2015.4.

(18) Comtet, J.; Glushkov, E.; Navikas, V.; Feng, J.; Babenko, V.; Hofmann, S.; Watanabe, K.; Taniguchi, T.; Radenovic, A. Wide-Field Spectral Super-Resolution Mapping of Optically Active Defects in Hexagonal Boron Nitride. *Nano Lett.* **2019**, *19* (4), 2516–2523. https://doi.org/10.1021/acs.nanolett.9b00178.
