# Supplementary figures and images for: Monitoring Electrochemical Dynamics through Single-Molecule Imaging of hBN Surface Emitters in Organic Solvents
Source: ACS Nano. 2024 Sep 25;18(40):27401–10. doi: 10.1021/acsnano.4c07189 (PMC11468151; doi:10.1021/acsnano.4c07189)

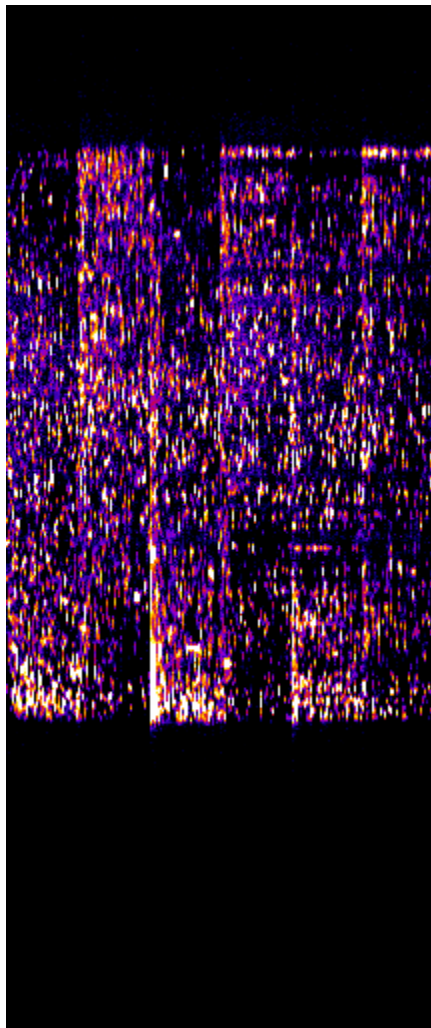

75x180mm (72 x 72 DPI)

Supplement: Supplementary file 4 — nn4c07189_si_004.pdf [file nn4c07189_si_004.pdf]
